# Protein Extraction, Enrichment and MALDI MS and MS/MS Analysis from Bitter Orange Leaves (*Citrus* *aurantium*)

**DOI:** 10.3390/molecules25071485

**Published:** 2020-03-25

**Authors:** Donatella Aiello, Carlo Siciliano, Fabio Mazzotti, Leonardo Di Donna, Roberta Risoluti, Anna Napoli

**Affiliations:** 1 Department of Chemistry and Chemical Technologies, University of Calabria, 87036 Arcavacata di Rende, Italy; donatella.aiello@unical.it (D.A.); fabio.mazzotti@unical.it (F.M.); l.didonna@unical.it (L.D.D.); 2 Department of Pharmacy, Health and Nutritional Sciences, University of Calabria, 87036 Arcavacata di Rende, Italy; carlo.siciliano@unical.it; 3 Department of Chemistry, Università degli Studi di Roma La Sapienza, 00185 Rome, Italy; roberta.risoluti@uniroma1.it

**Keywords:** *Citrus aurantium*, vacuum infiltration centrifugation, MALDI MS/MS, GDSLs, SPE enrichments

## Abstract

*Citrus aurantium* is a widespread tree in the Mediterranean area, and it is mainly used as rootstock for other citrus. In the present study, a vacuum infiltration centrifugation procedure, followed by solid phase extraction matrix-assisted laser desorption ionization tandem mass spectrometry (SPE MALDI MS/MS) analysis, was adopted to isolate proteins from leaves. The results of mass spectrometry (MS) profiling, combined with the top-down proteomics approach, allowed the identification of 78 proteins. The bioinformatic databases TargetP, SignalP, ChloroP, WallProtDB, and mGOASVM-Loc were used to predict the subcellular localization of the identified proteins. Among 78 identified proteins, 20 were targeted as secretory pathway proteins and 36 were predicted to be in cellular compartments including cytoplasm, nucleus, and cell membrane. The largest subcellular fraction was the secretory pathway, accounting for 25% of total proteins. Gene Ontology (GO) of *Citrus sinensis* was used to simplify the functional annotation of the proteins that were identified in the leaves. The Kyoto Encyclopedia of Genes and Genomes (KEGG) showed the enrichment of metabolic pathways including glutathione metabolism and biosynthesis of secondary metabolites, suggesting that the response to a range of environmental factors is the key processes in citrus leaves. Finally, the Lipase GDSL domain-containing protein GDSL esterase/lipase, which is involved in plant development and defense response, was for the first time identified and characterized in *Citrus aurantium*.

## 1. Introduction

Citrus species constitute one of the major tree fruit crops with great economic impact and is currently facing biotic and abiotic stresses. Rootstocks play a pivotal role in the success of the global commercial production of citrus fruits. The choice of rootstocks is of critical importance because their possibly unsatisfactory characteristics can cause serious failure in the citrus industry. In many instances, citrus rootstocks are the sole determining element that allows citrus to be grown in particular circumstances; they adapt trees to the effects of biotic and abiotic stresses and lead to excellent yields of high-quality fruits. Rootstocks are responsible for the ground anchoring and the proper development of trees, including water and nutrient absorption; they act as an energy source, provide carbohydrate storage, control the harvest time and fruit quality, ensure protection against soil borne diseases, and allow the adaptation of plants to soil and atmospheric conditions. Notwithstanding, every rootstock has one or more undesirable traits that preclude its universal use [1,2]. Those limitations have generally been highlighted from experimental data; alternatively, they have been described only after developing commercial experiences. Today, research projects are principally focused on the selection and preparation of new suitable citrus rootstocks; the improvement of their productivity and resistance characteristics remains the main objectives of the investigation. As a consequence, the study of plant proteome is fundamental in understanding protein differential expression and biological functions. Evidence led to the design of effective sample preparation methods for numerous plant species, tissues [3,4,5,6,7,8,9,10,11,12,13,14,15,16,17,18,19,20,21], and citrus leaves among others [22,23,24,25,26,27]. The rapid and significant upgrading of sensitivity, throughput and mass accuracy of modern mass spectrometers drastically improved gel-free proteomic approaches [28,29,30]. Mass spectrometry methodologies are rapid and sensitive tools for the identification [31] and quantitation [32,33] of metabolites, amino acids, proteins and their post-translational modifications [34,35]. Matrix-assisted laser desorption ionization mass spectrometry (MALDI MS), and tandem mass spectrometry (MS/MS) techniques are used as an alternative to Liquid Chromatography Electrospray Ionization (LC-ESI), for the highly sensitive analysis of low and high molecular weight compounds in complex matrices [36,37,38,39,40]. MALDI MS technique offers great advantages, such as short analysis times, high sensitivity, tolerance to contaminants, the ability to detect different components in highly complex mixtures, and the possibility to be combined with a rapid and simple preparation of the sample, preventing any possible analyte loss [41].

In this study, a simple procedure based on the vacuum infiltration and centrifugation with salt solutions, fractionation and trypsin digestion, followed by MALDI time of flight (TOF)/TOF mass spectrometry is applied to leaves of *Citrus aurantium*, an economically important fruit tree in the Mediterranean area, widely used as a rootstock for citrus. Vacuum infiltration with an extraction solution was adopted because it is applicable to small samples and allows the extraction of proteins reducing the contamination by non-protein components. The results of mass spectrometry (MS) profiling, combined with the top-down proteomics approach, allowed the identification of 78 proteins with a significant match. MS data were processed by the amino-acid sequence-based predictors TargetP, SignalP, ChloroP, WallProtDB and mGOASVM-Loc in order to establish the subcellular locations of the extracted proteome. Gene Ontology annotation of the *Citrus sinensis* genome was used to facilitate the functional annotation of the proteins that were identified in citrus. The main metabolic pathways including glutathione metabolism and biosynthesis of secondary metabolites were enriched suggesting that the response to a range of environmental factors is one of the key processes in citrus leaves. GDSL esterase/lipase variants (A0A067EBP6, A0A067EBA9, A0A067EF15, A0A067ENI5, A0A067EMQ7, and V4TXR3) and hydrolytic enzymes with multifunctional properties previously undescribed for citrus species, were characterized.

## 2. Results

### 2.1. Protein Identification

**Figure 1:**
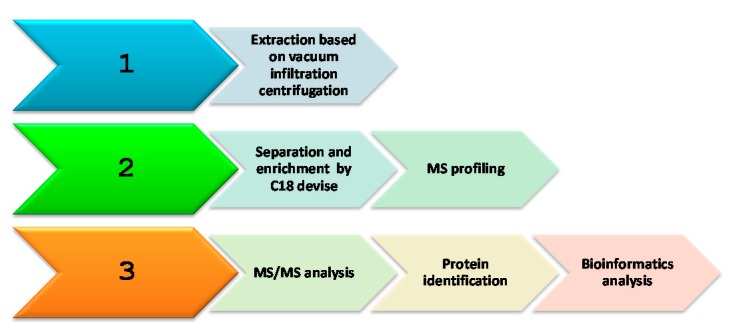
Schematic overview of the workflow.

Figure 1 displays the strategy adopted in this study. The experimental procedure was divided in three stages: protein extraction, separation, and identification of proteins by MS analysis combined with bioinformatics.

**Figure 2:**
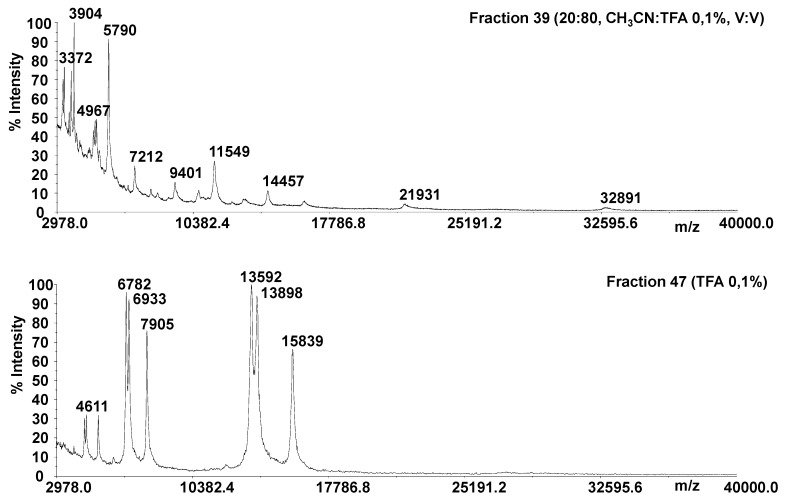
Linear matrix-assisted laser desorption ionization mass spectrometry (MALDI MS) of the chromatographic fractions **39** and **47**.

Many methods which are generally used to extract proteins from plant tissues have been based on a vacuum infiltration centrifugation together with an extraction solution process, followed by centrifugation [21,42]. We used a classical vacuum infiltration centrifugation method, slightly modified according to the description provided in the Materials and Methods section. It is known that proteins can be selectively solubilized depending on the chemical used for the extraction. The use of salt solutions is a commonly accepted tool [43,44], likewise NaCl has proven to be more effective in releasing the greatest number of proteins [43,44]. We applied a single washing step to obtain a protein fraction that was chromatographically fractionated by adopting a C18 cartridge. All chromatographic fractions were monitored by linear MALDI MS, in order to evaluate the intact protein mass information. Representative MALDI MS spectra are reported in Figure 2 (fractions **39** and **47**; Figure S1). Peak overlapping and charge state ambiguity occur to some extent in a top-down analysis of intact proteins using MALDI TOF-TOF platforms. In fact, mono and multicharged protein ions (+1, +2, +3, +4 and +6) were detected in several fractions. The protein precursor ions and the dissociation method employed affects the structural information that can be produced in a MS/MS experiment. The dissociation of intact proteins is a more difficult process than the peptide fragmentation. Top-down protein identification by database search based on peptide sequence tags from the MS/MS spectrum has been reported only for platforms displaying high resolving power [45,46,47,48,49,50,51,52,53]. Several approaches have been applied to obtain primary structure information from entire protein ions for proteins with molecular weights as large as several tens of kilodaltons [45,46,47,48,49,50,51,52,53]. The ions observed for the unknown proteins from fraction **47** were 41 kDa, 40 kDa (which are the calculated average mass from +3 and +6 protein ions) and 31kDa (from +2 and +4 protein ions, Figure 2). The electrophoretic profile resulting from fraction **47** highlighted the presence of two protein bands within 30–44 kDa (Figure S2). Therefore, the protein profile displayed by linear MALDI experiments agreed with that obtained by SDS-PAGE. Sequence information for the unknown proteins were obtained by digesting all fractions and performing MS/MS experiments on the digestion products. MS data obtained from a typical digested fraction, e.g., fraction **47**, were directly subjected to the National Center for Biotechnology Information (NCBI) database for protein identification against other green plants.

The database output allowed to identify a lipase-GDSL, by using 11 masses corresponding to six possible peptide sequences (gi|641833486, gi|641833485, gi|641833487, gi|568850564, gi|567901604, gi|641833488), characterized by significant protein scores (>60).

Therefore, spectral data collected from MS/MS experiments performed on all digested chromatographic fractions were subjected to a database search (Protein Pilot software) for the identification of proteins. A total of 78 proteins, belonging to *Citrus aurantium* and *Citrus sinensis* species, were identified with a significant match (Table 1). MS and MS/MS searches were performed against *Citrus aurantium* [43165] and *Citrus sinensis* [2711] protein sequence database, including sequences derived from SwissProt and TrEMBL (Translated EMBL Nucleotide Sequence Data Library). Alanine-tRNA ligase (A0A067FLL5, Table 1, row 1), Glutathione S-transferase (Q3HM93, Table 1, row 4), non-specific serine/threonine protein kinase (A0A067F884, Table 1, row 5), and RING-type E3 ubiquitin transferase (A0A067GDZ1, Table 1, row 7) represent the only named, however unreviewed MS/MS identified sequences.

### 2.2. Bioinformatic Analysis

#### 2.2.1. Prediction of Biological Processes and Protein Class

**Figure 3:**
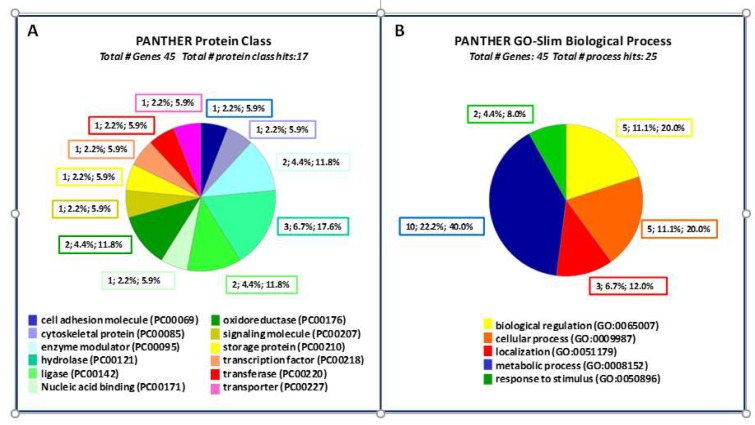
PANTHER functional classification viewed in pie chart. (**A**) Protein Class; (**B**) Biological Process.

The proteomics generated dataset (Table 1) was sorted into 12 different functional classes, cell adhesion molecule, cytoskeletal protein, enzyme modulator, hydrolase, ligase, nucleic acid binding, oxidoreductase, signaling molecule, storage protein, transcription factor, transferase and transporter. The dataset was analyzed using the genome of *Citrus sinensis* as the reference. PANTHER classification resulted in 45 gene entries which were analyzed for functional classification (Figure 3).

#### 2.2.2. Subcellular Localization Prediction

The subcellular localization of a protein is an important step in understanding its function. In this study TargetP, SignalP, ChloroP, WallProtDB and mGOASVM-Loc were used to predict the subcellular localization of the 78 identified proteins. The FASTA format of all proteins identified using *Citrus aurantium* [43165] and *Citrus sinensis* [2711] database was used for bioinformatic analysis.

The following strategy was adopted to distinguish the subcellular localization: (a) proteins having a signal peptide predicted by TargetP and SignalP were gathered in “secretory pathways”, (b) TargetP was used to predict the mitochondrial and chloroplast localization, (c) mGOASVM-Loc (Multi-Label Protein Subcellular Localization) was used for multi-location proteins, and (d) WallProtDB was used as a database resource for plant cell wall proteomics. The identified proteins (Table 1) were classified for their subcellular localization as deduced by TargetP1.1 [54]. The location assignment is based on the predicted presence of any of the N-terminal pre-sequences: chloroplast transit peptide (cTP), mitochondrial targeting peptide (mTP), or secretory pathway signal peptide (SP). TargetP output revealed 4 proteins containing a chloroplast transit peptide (cTP), 16 proteins containing secretory pathway signal peptides (SP), and 56 proteins were labeled as “other”, choosing specificity > 0.95 (cut-off restrictions were set as follows: 0.730 (cTP), 0.860 (mTP), 0.430 (SP) and 0.840 (other), respectively). The presence of a SP in a protein is considered as the signature of a secretory protein because much of the secretory protein is translocated across the endoplasmic reticulum (ER). Secretory proteins are directed to the ER membrane by an N-terminal signal peptide and are translocated by the same mechanism. Since TargetP also predicted proteins containing a chloroplast transit peptide (cTP), the FASTA formats of all proteins were submitted to ChloroP for a detailed report of the cTP scores along the sequences (Table S1).

The ChloroP output highlighted seven proteins (A0A067GUC9, A0A067GUN6, V4TXR3, A0A067EZE8, A0A067ECH7, A0A067EJ84, and A0A067FBM6) containing a cTP (Table S1). The predicted TargetP results were selected, and since this database can deal with multi-location proteins, the localization generated by mGOASVM-Loc was accepted. The proteins did not univocally assign to a compartment and so they were categorized as uncertain due to the discrepancies among the five programs. Finally, SignalP, TargetP, mGOASVM-Loc and WallProtDB tools for proteomics-generated data sets revealed the presence of eight subcellular fractions, including secretory pathway (20), cytoplasm (16), nucleus (8), cell membrane (6), chloroplast (4), endoplasmic-reticulum (3) peroxisome (1) and uncertain (29) (Table S1). According to the annotation protein function in Prosite (https://prosite.expasy.org), 20 proteins localized in the secretory pathway were sorted into nine groups: peptidase family A1, membrane lipoprotein, aspartyl proteases, sugar transport, soluble glutathione S-transferase N-terminal, soluble glutathione S-transferase C-terminal, sugar transport proteins, specific tyrosine protein kinases, and protein kinases (Table S2).

#### 2.2.3. Pathways Enrichment Analysis

**Figure 4:**
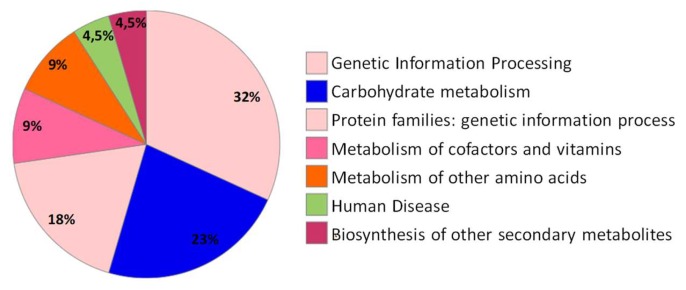
The protein sequences of citrus-specific genes were functionally annotated with metabolic information from the Kyoto Encyclopedia of Genes and Genomes (KEGG) pathway database using KEGG Orthology And Links Annotation (BlastKOALA) program.

The domains are the structural and functional units of proteins and can be used to assign an undescribed sequenced protein to a specific family of proteins, and to further formulate hypotheses about its function (Figure 4).

A critical step in understanding protein functions is the identification of relevant protein–protein interactions, such as the direct physical binding, indirect interaction and participation in the same metabolic pathways or cellular processes. Protein association network analysis is usually performed by the STRING database (Search Tool for the Retrieval of Interacting Genes/Proteins) [55]. This database includes interactions which have been described in the literature on the basis of experimentally studied relationships, as well as those obtained from the genome analysis performed using several methods that establish domain fusion, phylogenetic profiling and gene neighborhood concepts. Accordingly, a confidence score for every protein–protein association is assigned to the network. Higher scores designate an association supported by several types of evidence. In the present investigation, STRING analysis was exploited on the organism *Citrus sinensis*, using the list of 78 protein annotations (accession number from UniProtKB) reported in Table 1. STRING associated only 37 proteins matching the input list and failed the analysis, since the data set probably is a random collection of proteins that are not very well connected. This does not necessarily mean that it is not a biologically meaningful selection of proteins, but it could simply be that these proteins have not been studied and that their interactions might not yet be known to STRING.

BlastKOALA (Basic Local Alignment Search Tool-KEGG Orthology and Links Annotation, http://www.kegg.jp/blastkoala/) is automatic annotation server for genome and metagenome sequences, which perform KO (KEGG Orthology) assignments to characterize individual gene functions and reconstruct KEGG pathways. The 78 unique protein identifiers (Table 1, ID) were subjected to BlastKOALA [56] to obtain their corresponding K-numbers to further investigate the biological function of the proteins in the citrus leaves. Of the 78 entries, 22 entries (28.2%) were annotated. This provided a list of 22 unique K numbers that was then used for Kyoto Encyclopedia of Genes and Genomes (KEGG) mapping [57]. The K number assigned sequences were categorized according to the KEGG Orthology system (ko00001). The highlighted functional categories of annotated genes according to the KO system were genetic information processing (7 entries), carbohydrate metabolism (5 entries), protein families: genetic information processing (4 entries), metabolism of other amino acids (2 entries), metabolism of cofactors and vitamins (2 entries), human diseases (1 entries) and biosynthesis of other secondary metabolites (1 entries) (Figure 4). These 22 K numbers were mapped to 18 KEGG pathways (Tables S3 and S4) and 5 modules. The main pathways were “Metabolic pathways—*Citrus sinensis* (Valencia orange)” (score 81), “glutathione metabolism—*Citrus sinensis* (Valencia orange)” (score 58) and “biosynthesis of secondary metabolites—*Citrus sinensis* (Valencia orange)”(score 23).

#### 2.3. GDSL Esterase-Lipase Characterization

**Figure 5:**
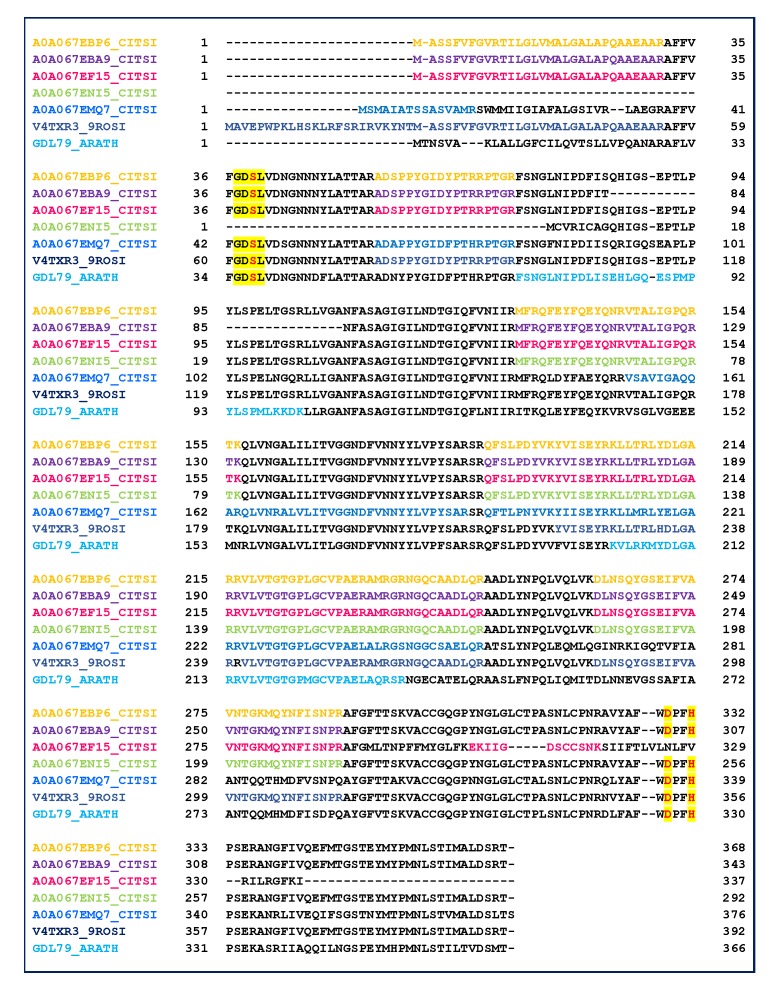
Alignment of the six identified GDSL variants with the sequence of A. *Thaliana* (GDL79_ARATH). MS/MS validated sequences are colored, while the yellow highlight the GDSL motif and the active sites (red amino acids).

**Figure 6:**
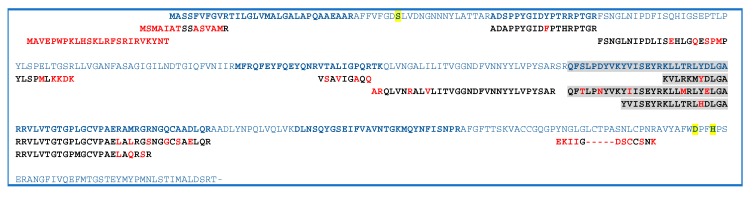
Sequence of GDSL from citrus by alignment of MS/MS validated peptides, using as reference sequence A0A067EBP6_CITSI (blue string). In bold is reported the validated sequences, in red is indicated the punctual modifications and in yellow is highlighted the catalytic triad, while the gray highlights the region with the major microheterogeneity of the protein.

The data reported in Table 1 highlight that several proteins belong to the GDSL lipase family (Table 1, lanes 61–66, gray region). GDSL lipases have been found in various plant species, including Arabidopsis *thaliana*, rice and maize, and their roles in plant development, morphogenesis and the defense response have been described [58,59]. Therefore, to improve the sequence coverage and characterization of proteins, all tandem mass spectra recorded for the single fraction 47 were evaluated by the MASCOT database searching. The oxidation of methionine and acetylation of protein N-term were also taken in account as the variable modifications. The results were carefully validated by a manual check of the corresponding MS/MS spectra. Six isoforms were recognized by direct submission of MALDI MS/MS data for protein identification. Table 2 shows collectively the peptide sequences produced by trypsin digestion and useful in identifying GDSL family. The alignment of the six identified GDSL variants with the GDSL sequence of A. *thaliana* (GDL79_ARATH) is reported in Figure 5. The MS/MS identified regions are colored, red used for the catalytic sites, and yellow for the GDSL motif. GDSL lipases represent a subfamily of lipolytic enzymes and possess a conserved catalytic triad (Ser, Asp, and His) [60]. However, unlike lipases that commonly contain a GxSxG motif, GDSL lipases exhibit a GxSxxxxG motif, in which the active site Ser is located near the N–terminus [60]. The alignment of peptides identified by MS/MS allowed to validate the expressed protein sequence (Figure 6, Table S5). The main protein microheterogeneity region suggested four isoforms to be present in the sample. Catalytic sites are not included in the identified peptides, and their positions are deduced only from the alignment with the validated sequence of A. *thaliana*. The prediction of protein functions and/or functional domains by bioinformatics tools is commonly used to classify an unknown. In these cases, the assumption is that proteins sharing functional domains have the same activity. The results obtained by comparing amino acid sequences (A0A067EBP6, A0A067EBA9, A0A067EF15, A0A067ENI5, A0A067EMQ7, V4TXR3) were combined with searches for functional domains (http://www.ebi.ac.uk/InterProScan/).

The predicted functional class was GDSL lipase/esterase-like (IPR035669), a plant specific subfamily of the SGNH-family of hydrolases, acting on ester bonds. The SGNH hydrolase superfamily represents a subgroup of the GDSL family, based on the presence of four residues Ser, Gly, Asn, and His which are present in four conserved regions (blocks I, II, III, and V, respectively). This subgroup of enzymes has been found to be secreted and involved in the response to stimuli [61]. The signal peptide indicated in UniProt as 1–28 for the sequences A0A067EBP6_CITSI, A0A067EBA9_CITSI, A0A067EF15_CITSI, confirmed the secreted nature of those proteins.

## 3. Discussion

In the present study, a MS-based proteomic analysis was used for the analysis of leaves from *Citrus aurantium*, growing under normal conditions. A total of 78 proteins belonging to citrus species were identified through proteomics-generated data sets. MS and MS/MS searches were performed against the *Citrus aurantium* [43165] and *Citrus sinensis* [2711] protein sequence database, including sequences derived from SwissProt and TrEMBL (Translated EMBL Nucleotide Sequence Data Library). Although, the *Citrus aurantium* database is the most appropriate for identifying species-specific gene products, it suffers from the inherent limitation due to reviewed sequences (only 101 entries). To overcome this limitation and to expand the dataset of the identified proteins, the TrEMBL [43064 entries] database research was performed.

Among the 78 identified only four proteins are “named” but “unreviewed”: Alanine-tRNA ligase (A0A067FLL5, Table 1, row 1), Glutathione S-transferase (Q3HM93, Table 1, row 4), non-specific serine/threonine protein kinase (A0A067F884, Table 1, row 5), and RING-type E3 ubiquitin transferase (A0A067GDZ1, Table 1, row 7). Alanine-tRNA ligase (A0A067FLL5, Table 1, row 1) catalyzes the attachment of alanine to tRNA. Literature data reported RNA ligases to be active in vitro on a variety of substrates, and capable of inter- and intra-molecular RNA joining. Their role in vivo might comprise yet unknown essential functions aside from their involvement in pre-tRNA splicing [62]. Glutathione S-transferase (GST, Q3HM93, Table 1, row 4) is involved in the metabolic process of transport and/or accumulation of both anthocyanins and proanthocyanidins in the vacuole, that are well known plant pigments sharing common flavonoid intermediates until the formation of anthocyanidins. Literature data on A. *thaliana* report that the GST binding activity is affected by a single amino acid substitution. GST overexpression has been found to enhance the growth of transgenic tobacco seedlings during stress [63,64]. The non-specific serine/threonine protein kinase (A0A067F884, Table 1, row 5) plays an important role in the plant defense response in A. *thaliana* [65,66]. The RING-type E3 ubiquitin transferase (A0A067GDZ1, Table 1, row 7) regulates the defense response of a plant to pathogenic agents. E3 ubiquitin ligase activity is correlated to the cell death and defense in Solanaceae and Brassicaceae, as reported in the literature [67].

The list of MS/MS identified proteins (Table 1) does not represent the whole predicted proteome of citrus leaves. There are several reasons that can limit the coverage of proteome observed for *C. aurantium* leaves. It must be underlined that the mild extraction procedures employed did not allow extracting highly hydrophobic proteins, which may be considered a limitation of this approach. It could be thought that some proteins were probably present at concentrations that might be undetectable by the currently employed separation and sequencing techniques, although the highly sensitive MALDI TOF/TOF platform used in this investigation can generally enable the detection of very low amounts of analytes (10 pmol/μL).

According to SignalP, TargetP, mGOASVM-Loc, and WallProtDB the proteomics-generated data set (Table 1) was sorted into eight subcellular fractions, recognized as secretory pathway, cytoplasm, nucleus, cell membrane, chloroplast, endoplasmic-reticulum, peroxisome and “uncertain”. The largest subcellular fraction was the secretory pathway, accounting for 25% of total proteins. However, only 11 of the 20 secretory pathway proteins were indicated as cell wall proteins, according to CellWallDB. Recently, it has been reported that proteins present in the cell wall, lacking a signal peptide, may be excluded via more than a single non-classical secreted mechanism, such as secretory exosomes, lysosomes membrane, transporting and unknown [44,68,69,70]. In fact, proteins A0A067DDE4 and A0A067EPP0 (Table 1, row 13 and 30) were also retrieved in WallCellDB although they did not hold a signal peptide. A0A067EPP0 (LRR receptor-like serine threonine-protein kinase) and A0A067DDE4 (protein kinase) are two protein kinase domain-containing proteins (Table S2) found in grapevine [71] and thought to be involved in the development and stress responses. According to the annotate protein function in Prosite (https://prosite.expasy.org), 20 proteins localized in the secretory pathway were sorted into nine groups: peptidase family A1, membrane lipoprotein, aspartyl proteases, sugar transport, soluble glutathione S-transferase N-terminal, soluble glutathione S-transferase C-terminal, sugar transport proteins, specific tyrosine protein kinases, and protein kinases (Table S2). The role and the biological functions of the proteins belonging to peptidase family A1 and aspartyl proteases are still hypothetical. These proteases are involved in protein processing and/or degradation in different plant organs, as well as in plant senescence, stress responses, programmed cell death and reproduction.

The MS-based approach was also successfully used for the identification of six isoforms of GDSL (A0A067EBP6, A0A067EBA9, A0A067EF15, A0A067ENI5, A0A067EMQ7, V4TXR3), displaying point mutations in the region 186–214, as is well established by MS/MS experiments. The characterization of these stress responsive hydrolytic enzymes in C. *aurantium* is here reported for the first time. A GDSL-lipase family protein, called GLIP, has previously been identified as stress responsive secreted proteins in Arabidopsis *thaliana* [72]. This subclass of lipolytic enzymes has been related to seed development, lipid metabolism [73], and cutin formation [74]. Studies focusing on the secretion of GDSL-lipase family proteins have suggested the multiple functions that these enzymes exert in plants under normal growth and stress conditions [43,44].

GO enrichment analysis highlight some features of leaves proteome. Firstly, the main metabolic pathways including glutathione metabolism and biosynthesis of secondary metabolites were enriched suggesting that the response to a range of environmental factors is the key processes in citrus leaves. Plants deploy secondary metabolites to assist the interactions with the biotic and abiotic environment, including the essential role of chemical defense against herbivores and pathogens. The deployment of secondary metabolites, i.e., molecules that have no direct role in the primary functions, depends on genetic variability and can also be modified in response to environmental factors [75]. Glutathione metabolism is also correlated to the plant defense system and is directly linked to sulfur metabolism. Holler reported a link between the activation of cysteine and glutathione metabolism with sulfur-induced resistance in tobacco plants [76]. In particular, glutathione is known to be involved in plant defense reactions as a signaling molecule, and it has also been reported to cross-communicate with other established signaling molecules [76]. The key enzyme of pathways is Glutathione S-transferase (GST, EC. 1.1.18). GSTs represent a multifunctional family of enzymes may be involved in the conjugation of reduced glutathione to a wide number of exogenous and endogenous hydrophobic electrophiles. Evidence suggests that GSTs play an important role in the detoxification of both endogenous and xenobiotic compounds, and they are also involved in intracellular transport, bio-synthesis of hormones, and protection against oxidative stress [77,78,79,80,81]. The analysis of the gene expressions in orange leaf indicated that the isoforms GSTU1, and GSTU2 are distinctly expressed in the leaf [82] It was also showed that the expression of U1 gene was remarkably induced in response to stress while the U2 isoform was constitutively expressed playing some sort of“ default scavenging” activity in vivo. GSTs provide a tool to control weeds in agronomic crops [83,84]. The overexpression of heterologous GST genes is widely related to enhance the crop qualitative and quantitative features. The antioxidant activity of GST limits the damages and the extent of programmed cell death during the hypersensitive response. In particular, the GST expression is up-regulated during the resistance process against pathogenic attack and represents a positive regulator. Therefore, proteomic data reported here highlight that *Citrus aurantium* might be a rootstock with good features for the correct and optimal growth of citrus fruit trees.

## 4. Materials and Methods

### 4.1. Chemicals

Trifluoroacetic acid (TFA), methanol (MeOH), acetonitrile (ACN), H_2_O, acetone (CH_3_COCH_3_) ammonium bicarbonate (NH_4_HCO_3_, 99.5%), trypsin, α-cyano-4-hydroxy-trans-cynnamic acid (α-CHCA, 99.0%) and sodium chloride (NaCl, ≥ 99%) were purchased from Sigma-Aldrich (Italy).

### 4.2. Plant Materials

Experiments were carried out on leaves from three *Citrus aurantium var. amara* plants. To minimize errors, three biological repeats were conducted for proteome analysis. For each biological repeat sample, ten leaves from 3 *Citrus aurantium* plants were pooled. All experiments were repeated in three independent times, resulting in three technical and three biological replicates. Plants were grown in a botanical garden (Orto Botanico, 964H + QJ Arcavacata, Rende CS) under natural conditions. Standard cultural practices included drip irrigation. Drip irrigation frequency was modified to seasons and ranged from once weekly (winter) to five days/week (summer), with 40 L tree-1 per irrigation. Leaves were harvested during October 2018 (wet season). The age of the plants was 3 years.

### 4.3. Protein Extraction

*Citrus aurantium* leaves (5 g) were washed with deionized water and then cut into segments. Leaf segments (5 cm) were placed in a centrifuge tube and added with 15 mL of NaCl 50 mM to extract proteins by constant horizontal shaking (200 rpm) for 1 h on ice, followed by vacuum-infiltration and centrifugation at 1500× *g* for 10 min at 4 °C. Thereafter, the supernatant was added with 5 mL of CH_3_COCH_3_ vortexed for 10 min. After centrifugation (1500× *g*, 5 min), the organic solvent was removed under nitrogen flow, the aqueous proteins solution was reduced to 4 mL in a vacuum centrifuge (Speed-Vac, Cryo Rivoire) and stored at −20 °C until analysis. Protein concentration (100 μg/mL) was determined using the UV-160 spectrophotometer (Shimadzu, Kyoto, Japan) by the Bradford method. Bovine Serum Albumin (BSA) was used as the standard.

### 4.4. Solid Phase Extraction (SPE) Procedures

Protein extract was pre-purified by SPE (55 um, 70 A, Phenomenex, USA) equilibrated with acidified water (0.1% trifluoroacetic acid, TFA). Four milliliters of protein extract (100 μg/mL) were added with 2 µL of TFA 2% and loaded and washed with 1ml water. Elutions were performed with 20%, 40%, 60% and 80% acetonitrile in acidified water (4 mL for each step) [85]. All fractions were freeze-dried in a vacuum centrifuge (Speed-Vac, Cryo Rivoire) and subsequently reconstituted with NH_4_HCO_3_ (50 mM) at 1/10 of the initial volume. An aliquot (1 μL) of each fraction was analyzed by MALDI MS in linear mode. Fractions showing the same MALDI protein profile were unified.

### 4.5. SDS PAGE

A series of SPE fractions were separated by one-dimensional SDS-PAGE (Electronic Supplementary Material, Figure S1 ESM). Each SPE fraction (≈ 8 μg) was mixed with 5× gel loading buffer, containing 2-mercaptoethanol and bromophenol blue, denatured at 95 °C for 10 min before electrophoresis analysis in 12.5% sodium dodecyl sulphate-polyacrylamide gel electrophoresis. A homemade protein molecular weight marker (Lactoferrin 87 kDa, L9507; Bovine Serum Albumin 66 kDa, A2153; Albumin from chicken 44 kDa, A5503; Mioglobin from equine skeletal muscle 17 kDa, M0630; Cytocrome C 12 kDa, C2506) was loaded in the molecular weight marker lane. Proteins were stained with Comassie Brillant Blu R-250 for 4 h and destained overnight with a solution containing 40% MeOH, 10% CH_3_COOH and 50% H_2_O.

### 4.6. In-Solution Digestion

In-solution protein digestion was performed by adding 2 µL of trypsin (4 pmol/µL) to each chromatographic fraction. The complete microwave-assisted digestion was obtained after three treatments in the microwave (MWD 246 SL, Whirlpool Europe, Italy) at 250 W irradiation power each lasting for 3 min.

### 4.7. Mass Spectrometry Analysis

A 1 μL amount of each protein chromatographic fraction was mixed with 10 μL of α-CHCA (5 mg/mL). A 1 μL portion of sample−matrix solution was spotted on a MALDI matrix target, dried at room temperature, and directly analyzed by MALDI mass spectrometry. MS analyses were performed using a 5800 MALDI TOF/TOF analyzer (AB SCIEX, Germany) equipped with a neodymium: yttrium-aluminium-garnet laser (349 nm). Linear MALDI MS spectra were acquired averaging 4000 laser shots with a mass accuracy of 500 ppm in default calibration mode that was performed using the following set of standards: aldolase (rabbit, [M+H]+avg = 39905), BSA (bovin serum albumin [M+H]+avg = 66431) and IgG1 (murine myeloma [M+H]+avg = 148500).

Tryptic peptide solution (1 μL) was mixed with 10 μL of α-CHCA. A 1 μL portion of sample−matrix solution was spotted on a MALDI matrix target, dried at room temperature, and directly analyzed by MALDI mass spectrometry in reflectron positive mode with a mass accuracy of 5 ppm. Typically, 4000 laser shots were accumulated with a laser pulse rate of 400 Hz in the MS mode, whereas in the MS/MS mode spectra up to 5000 laser shots were acquired and averaged with a pulse rate of 1000 Hz. MS/MS experiments were performed at a collision energy of 1 kV, and ambient air was used as the collision gas with a medium pressure of 10−6 Torr. Protein identification was performed by the Protein Pilot 4.0 software program (AB Sciex) using the Paragon (AB Sciex) protein database search algorithm. The data analysis parameters were as follows: Sample Type: Identification; Cys Alkylation: None; Digestion: Trypsin; Instrument: 5800; Special factors: None; Species: None ID; Focus: Biological modifications—Amino acid substitution; Database: uniprot-taxonomy_Citrus + aurantium + (Bitter+orange) + (Citrus+vulgaris) + [43165]_.fasta and uniprot-taxonomy_ Citrus + sinensis + (Sweet + orange) + (Citrus+aurantium + var + sinensis) + [2711]_.fasta; Search Effort: Thorough ID; FDR analysis: Yes; Used Modified Parameter Files: No; Detected Protein Threshold [Unused ProtScore (Conf)]:1.5 (95.0%). Spectra were also handled using Data Explorer version 4.11 (AB Sciex). The MS/MS data were processed to assign candidate peptides in the NCBI and UniProt database using the MASCOT search program (http://www.matrixscience.com). The mass tolerance of the parent and fragments for MS/MS data search was set at 10 ppm and 0.20 Da, respectively. The query was made for “Other Green Plants” taxonomy allowing 2 missed cleavage. A Peak-list of 50 fragment ions of intensity higher than 10% above the noise level was generically used for the database search. All spectra were manually checked to verify the validity of the MASCOT results.

### 4.8. Database Proteomics, Targeting Predictions and Functional Classification

The presence and location of signal peptide cleavage sites are predicted by the SignalP 3.0 server, which contains two prediction programs (SignalP-HMM and Signal-NN; http://www.cbs.dtu.dk/services/SignalP) [54].

SignalP software (http://www.cbs.dtu.dk/services/SignalP), which searches for signal sequences and their cleavage sites, is generally used to determine whether identified secreted proteins contain signal peptides. TargetP [86] is applied to predict the mitochondrial localization and not to consider them as secreted proteins. Additionally, Multi-Label Protein Subcellular Localization Prediction (mGOASVM (V1), http://bioinfo.eie.polyu.edu.hk/mGoaSvmServer/mGOASVM_v1.html) [87] was adopted for general localization prediction purpose. Identification of conserved domains in identified proteins is performed using the Prosite (https://prosite.expasy.org).

Network analysis was performed submitting the orthologous Arabidopsis ID to the STRING (Search Tool for the Retrieval of Interacting Genes) software (v.11) (http://stringdb.org/) [55] Functional and Gene Ontology (GO) analysis was performed by PANTHER program (http://www.pantherdb.org/) [88] and BlastKOALA (Query dataset: 78 entries; Taxonomy group: Eukaryotes, Plants; KEGG database searched: family_eukaryotes.pep,genus_prokaryotes.pep; 22 entries (28.2%) annotated) [56,57].

## Figures and Tables

**Table 1:** Identified proteins by matrix-assisted laser desorption ionization tandem mass spectrometry (MALDI MS/MS) and Protein Pilot Paragon Method. The tandem mass spectrometry (MS/MS) data were processed using a mass tolerance of 10 ppm and 0.2 Da for the precursor and fragment ions, respectively.

	Accession ^a^	Protein Name ^a^	Specie ^a^	Functions and Domains ^a^	MW ^a^
1	A0A067FLL5	Alanine--tRNA ligase	*c. s.*	mitochondrial alanyl-tRNA aminoacylation, ATP binding and protein biosynthesis	80.538
2	A0A067FXS4	Alanine--tRNA ligase	*c. s.*	mitochondrial alanyl-tRNA aminoacylation, ATP binding and protein biosynthesis	85.228
3	A0A067EAN5	Belongs to the zinc-containing alcohol dehydrogenase family.	*c. s.*	oxidoreductase activity, zinc ion binding	40.377
4	Q3HM93	Glutathione S-transferase	*c. s.*	transferase activity	24.239
5	A0A067F884	Non-specific serine/threonine protein kinase	*c. s.*	ATP binding and protein serine/threonine kinase activity	49.609
6	A0A067FQ29	Probable alanine--tRNA ligase, chloroplastic	*c. s.*	Aminoacyl-tRNA synthetase, Ligase, RNA-binding, tRNA-binding	104.431
7	A0A067GDZ1	RING-type E3 ubiquitin transferase	*c. s.*	ubiquitin-protein transferase activity	78.497
8	A7U3F5	RNA polymerase B (Fragment)	*c. a.*	DNA binding	17.157
9	C6KK63	RpoB (Fragment)	*c. a.*	DNA binding	13.966
10	A0A067EE86	Similar to Putative alcohol dehydrogenase	*c. s.*	oxidation-reduction process	27.319
11	A0A067H5U9	Sodium/hydrogen exchanger	*c. s.*	sodium:proton antiporter activity	57.964
12	A0A067DAD8	Uncharacterized protein	*c. s.*	Hypotetical member of ribonuclease H-like superfamily	42.202
13	A0A067DDE4	Uncharacterized protein	*c. s.*	protein kinase activity	50.12
14	A0A067DMF5	Uncharacterized protein	*c. s.*	aspartic-type endopeptidase activity	37.197
15	A0A067DUQ6	Uncharacterized protein	*c. s.*	aspartic-type endopeptidase activity	42.258
16	A0A067DV99	Uncharacterized protein	*c. s.*	gene silencing by RNA, containig XS domain	132.604
17	A0A067DVX6	Uncharacterized protein	*c. s.*	aspartic-type endopeptidase activity	40.249
18	A0A067DXK8	Uncharacterized protein	*c. s.*	containing development and cell death domain	66.65
19	A0A067DYD3	Uncharacterized protein	*c. s.*	oxidation-reduction process	28.91
20	A0A067DYR7	Uncharacterized protein	*c. s.*	oxidation-reduction process	36.107
21	A0A067DZ88	Uncharacterized protein	*c. s.*		104.139
22	A0A067E608	Uncharacterized protein	*c. s.*	containing development and cell death domain	66.418
23	A0A067EAX4	Uncharacterized protein	*c. s.*	similar to Importin subunit alpha-6 (Arabidopsis thaliana), protein transporter activity	61.989
24	A0A067ECD2	Uncharacterized protein	*c. s.*	DNA binding	26.85
25	A0A067ECH7	Uncharacterized protein	*c. s.*	ATP binding and protein kinase activity	86.835
26	A0A067EGL9	Uncharacterized protein	*c. s.*	oxidoreductase activity	31.792
27	A0A067EJ07	Uncharacterized protein	*c. s.*	transcription factor activity, containig GATA-type domain	34.845
28	A0A067EJ84	Uncharacterized protein	*c. s.*	methyltransferase activity	38.231
29	A0A067EKU4	Uncharacterized protein	*c. s.*	DNA binding; protein containing SAND domain	20.855
30	A0A067EPP0	Uncharacterized protein	*c. s.*	ATP binding and protein kinase activity	113.792
31	A0A067ES66	Uncharacterized protein	*c. s.*	containing coiled coil domaina	55.599
32	A0A067EVC3	Uncharacterized protein	*c. s.*	metal binding, containig zinc finger (Znf) domains	31.557
33	A0A067F275	Uncharacterized protein	*c. s.*	similar to Glutathione S-transferase (C. S.)	24.233
34	A0A067FBM6	Uncharacterized protein	*c. s.*	transcription factor activity,	27.199
35	A0A067FNX1	Uncharacterized protein	*c. s.*		17.715
36	A0A067FS06	Uncharacterized protein	*c. s.*	containing 3 coiled coil domain	98.755
37	A0A067FYX5	Uncharacterized protein	*c. s.*	aspartic-type endopeptidase activity	41.826
38	A0A067FZS8	Uncharacterized protein	*c. s.*	protein serine/threonine phosphatase activity	64.983
39	A0A067G2U9	Uncharacterized protein	*c. s.*		54.933
40	A0A067G2Z9	Uncharacterized protein	*c. s.*		53.389
41	A0A067G6L7	Uncharacterized protein	*c. s.*	O-methyltransferase activity	105.692
42	A0A067G9E6	Uncharacterized protein	*c. s.*	O-methyltransferase activity	105.779
43	A0A067GBI2	Uncharacterized protein	*c. s.*	protein serine/threonine phosphatase activity	78.787
44	A0A067GET1	Uncharacterized protein	*c. s.*		50.073
45	A0A067GIB5	Uncharacterized protein	*c. s.*	DNA binding and regulation of transcription	31.651
46	A0A067GIK6	Uncharacterized protein	*c. s.*	O-methyltransferase activity	103.594
47	A0A067GIV0	Uncharacterized protein	*c. s.*	O-methyltransferase activity	86.016
48	A0A067GNR1	Uncharacterized protein	*c. s.*	ubiquitin-protein transferase activity	407.981
49	A0A067GQL4	Uncharacterized protein	*c. s.*		71.588
50	A0A067GRF1	Uncharacterized protein	*c. s.*	ubiquitin-protein transferase activity	395.412
51	A0A067GT43	Uncharacterized protein	*c. s.*	containing Cir_N domain and coiled coil doman	48.391
52	A0A067GUC9	Uncharacterized protein	*c. s.*	Potential transmembrane proteins	30.548
53	A0A067GUN6	Uncharacterized protein	*c. s.*	Potential transmembrane proteins	23.614
54	A0A067GV48	Uncharacterized protein	*c. s.*		83.17
55	A0A067GVN8	Uncharacterized protein	*c. s.*	DNA binding and regulation of transcription	27.869
56	A0A067GYR1	Uncharacterized protein	*c. s.*	containing post-SET domain	87.903
57	A0A067H0N2	Uncharacterized protein	*c. s.*	ubiquitin-protein transferase activity	406.782
58	A0A067H3Y3	Uncharacterized protein	*c. s.*	pyridoxal phosphate binding	51.821
59	A0A067GNF9	Uncharacterized protein	*c. s.*	ubiquitin-protein transferase activity	407.805
60	A0A067DIT7	Uncharacterized protein	*c. s.*	aspartic-type endopeptidase activity	45.31
61	A0A067EBP6	Uncharacterized protein	*c. s.*	hydrolase activity, acting on ester bonds. Belongs to the ‘GDSL’ lipolytic enzyme family. Signal Peptide (1-29).	40.484
62	A0A067EBA9	Uncharacterized protein	*c. s.*	hydrolase activity, acting on ester bonds. Belongs to the ‘GDSL’ lipolytic enzyme family. Signal Peptide (1-29).	37.88
63	A0A067EF15	Uncharacterized protein	*c. s.*	Signal Peptide (1-31); Lipase_GDSL domain (34 – 316. Hydrolase activity, acting on ester bonds. Belongs to the ‘GDSL’ lipolytic enzyme family.	37.337
64	A0A067ENI5	Uncharacterized protein	*c. s.*	Lipase_GDSL domain (78-265). Hydrolase activity, acting on ester bonds. Belongs to the ‘GDSL’ lipolytic enzyme family	32.421
65	A0A067EMQ7	Uncharacterized protein	*c. s.*	Lipase_GDSL domain (40 – 352). Hydrolase activity, acting on ester bonds. Belongs to the ‘GDSL’ lipolytic enzyme family	41.18
66	V4TXR3	Uncharacterized protein	*c. s*	Lipase_GDSL domain (58-365). Hydrolase activity, acting on ester bonds.	43.441
67	A0A067FW02	Uncharacterized protein	*c. s.*	Signal Peptide (1–20); Peptidase A1 domain (140-476). Aspartic-type endopeptidase activity. Belongs to the peptidase A1 family	50.918
68	A0A067FVB0	Uncharacterized protein	*c. s.*	Signal Peptide (1–20); Peptidase A1 domain (140–476). Aspartic-type endopeptidase activity. Belongs to the peptidase A1 family	48.178
69	A0A067DCQ1	Uncharacterized protein (Fragment)	*c. s.*	solute:proton antiporter activity	84.525
70	A0A067DDS7	Uncharacterized protein (Fragment)	*c. s.*		63.918
71	A0A067DW09	Uncharacterized protein (Fragment)	*c. s.*		13.294
72	A0A067DZ15	Uncharacterized protein (Fragment)	*c. s.*	diacylglycerol O-acyltransferase activity	50.24
73	A0A067ED32	Uncharacterized protein (Fragment)	*c. s.*	containing coiled coil domain	13.593
74	A0A067EZE8	Uncharacterized protein (Fragment)	*c. s.*	containing domain of unknown function (DUF1995)	36.936
75	A0A067FVE2	Uncharacterized protein (Fragment)	*c. s.*	containing 5 coiled coil domain	124.974
76	A0A067G352	Uncharacterized protein (Fragment)	*c. s.*	containing 5 coiled coil domain	121.041
77	A0A067GCY0	Uncharacterized protein (Fragment)	*c. s.*	microtubule binding	68.001
78	A0A067GQ70	Uncharacterized protein (Fragment)	*c. s.*	catalytic activity	38.307

^a^ According to “UniProtKB” (http://www.uniprot.org/), c.s.: *Citrus sinensis*, c.a.: *Citrus aurantium*.

**Table 2:** MS/MS identified peptides from GDSL esterase family by trypsin digestion.

Sequence ^a^	Mr found ^b^	Mr calc ^b^
YIISEYRK	1071.59	1071.58
QFSLPDYVK	1096.58	1096.57
QFTLPNYVK	1109.61	1109.60
MASSFVFGVR (1Acetyl)	1142.58	1142.57
mASSFVFGVR (1Acetyl)	1158.57	1158.56
GSNGGCSAELQR	1178.53	1178.52
VTALIGPQRTK	1183.73	1183.72
EKIIGDSCCSNK	1296.61	1296.59
KVLRKmYDLGAR	1465.85	1465.83
KLLmRLYELGAR	1478.87	1478.85
MSMAIATSSASVAMR	1513.73	1513.72
KLLmRLYELGARR	1634.97	1634.95
AMRGRNGQCAADLQR	1646.81	1646.80
VKYNTMASSFVFGVR	1705.89	1705.87
VSAVIGAQQARQLVNR	1709.99	1709.98
VLVTGTGPLGCVPAERAMR	1927.04	1927.03
1Met-loss (-)MAVEPWPKLHSKLRFSR	1951.12	1951.10
ADAPPYGIDFPTHRPTGR	1967.99	1967.97
AVEPWPKLHSKLRFSR (1Acetyl)	1993.13	1993.11
ADSPPYGIDYPTRRPTGR	2019.02	2019.00
RVLVTGTGPLGCVPAELALR	2022.17	2022.15
TILGLVmALGALAPQAAEAAR	2053.17	2053.15
RVLVTGTGPLGCVPAERAMR	2083.15	2083.13
QFTLPNYVKYIISEYRK	2162.19	2162.16
YVISEYRKLLTRLHDLGAR	2303.32	2303.30
RVLVTGTGPLGCVPAERAmRGR	2312.27	2312.24
YVISEYRKLLTRLYDLGAR	2329.33	2329.30
FSRIRVKYNTMASSFVFGVR	2365.28	2365.26
QFSLPDYVKYVISEYRKLLTR	2618.46	2618.43
ALVLITVGGNDFVNNYYLVPYSAR	2658.42	2658.39
MASSFVFGVRTILGLVmALGALAPQAAEAAR	3134.72	3134.68
mYDLGARRVLVTGTGPmGCVPAELAQRSR	3136.61	3136.58
MFRQFEYFQEYQNRVTALIGPQRTK	3150.63	3150.59
mASSFVFGVRTILGLVMALGALAPQAAEAAR (1Acetyl)	3176.73	3176.70
DLNSQYGSEIFVAVNTGKMQYNFISNPR	3192.57	3192.54
FSNGLNIPDLISEHLGQESPMPYLSPMLKKDK	3598.86	3598.83

^a^ Amino acid sequence of peptides identified from Trypsin digests on the basis of their CID spectra. ^b^ All mass values are listed as monoisotopic mass [M + H]^+^. m denotes methionine oxidized.

## Supplementary Materials

The following are available online at https://www.mdpi.com/1420-3049/25/7/1485/s1, Figure S1: Linear MALDI spectra of the chromatographic fractions. Figure S2: SDS-PAGE of fraction **47**. Table S1. Predicted subcellular localization of the 78 identified proteins. Table S2: PROSITE output for the 20 proteins localized in the secretory pathway. Table S3: K numbers (KO) by BlastKOALA (https://www.kegg.jp/blastkoala/). Table S4: KEGG Mapper Search Result. Table S5. MS/MS identified peptides of GDSLs.
