# Supplementary material for: Protein Extraction, Enrichment and MALDI MS and MS/MS Analysis from Bitter Orange Leaves (Citrus aurantium)
Source: Molecules. 2020 Mar 25;25(7):1485. doi: 10.3390/molecules25071485 (PMC7181213; doi:10.3390/molecules25071485)
Supplement: Supplementary file 1 [file molecules-25-01485-s001.pdf]

Supplementary material

Protein extraction, enrichment and MALDI MS and MS/MS analysis from bitter orange leaves (Citrus aurantium).

Donatella Aiello [1], Carlo Siciliano [2], Fabio Mazzotti [1], Leonardo Di Donna [1], Roberta Risoluti [3], Anna Napoli [1,*]

[1] Department of Chemistry and Chemical Technologies, University of Calabria, Italy.
[2] Department of Pharmacy, Health and Nutritional Sciences, University of Calabria, Italy.
[3] Department of Chemistry, Università degli Studi di Roma La Sapienza, Rome, Italy.

Figure S1: Linear MALDI spectra of the chromatographic fractions.

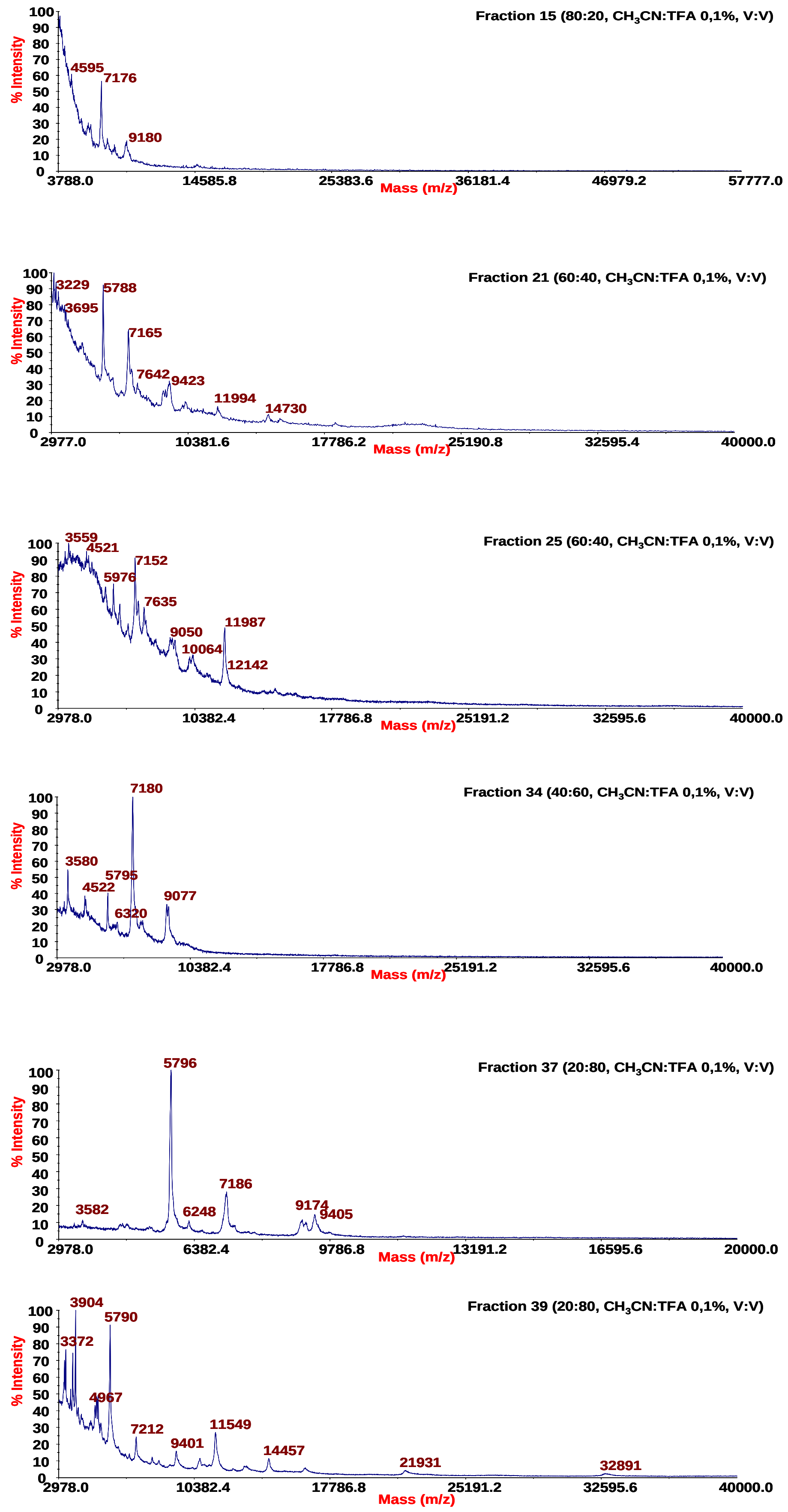

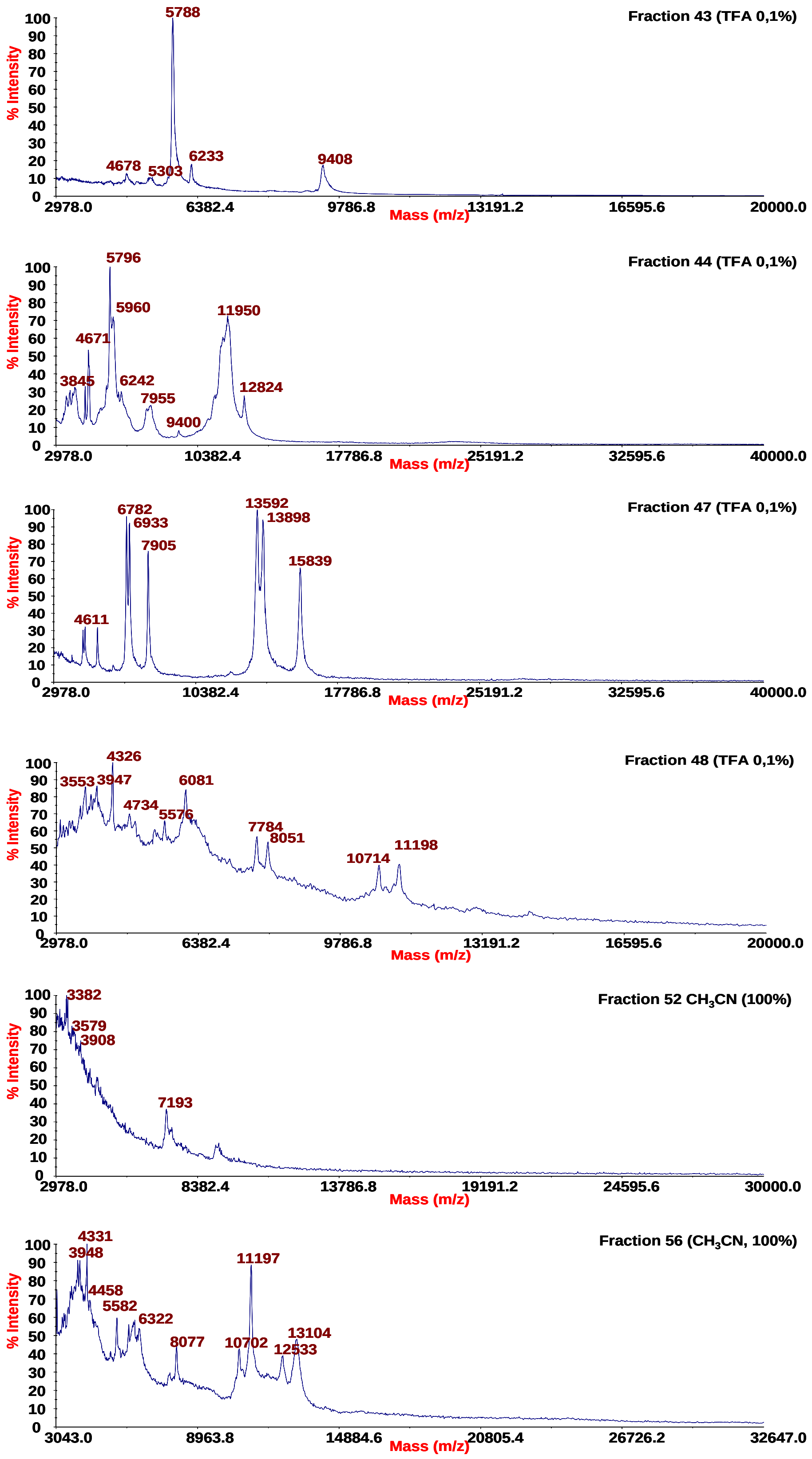

Fraction 43 (TFA 0,1%)
% Intensity
Mass (m/z)
5788
4678
5303
6233
9408
2978.0
6382.4
9786.8
13191.2
16595.6
20000.0
Fraction 44 (TFA 0,1%)
% Intensity
Mass (m/z)
5796
5960
4671
3845
6242
7955
9400
11950
12824
2978.0
10382.4
17786.8
25191.2
32595.6
40000.0
Fraction 47 (TFA 0,1%)
% Intensity
Mass (m/z)
6782
6933
7905
4611
13592
13898
15839
2978.0
10382.4
17786.8
25191.2
32595.6
40000.0
Fraction 48 (TFA 0,1%)
% Intensity
Mass (m/z)
4326
3553
3947
4734
5576
6081
7784
8051
10714
11198
2978.0
6382.4
9786.8
13191.2
16595.6
20000.0
Fraction 52 CH3CN (100%)
% Intensity
Mass (m/z)
3382
3579
3908
7193
2978.0
8382.4
13786.8
19191.2
24595.6
30000.0
Fraction 56 (CH3CN, 100%)
% Intensity
Mass (m/z)
4331
3948
4458
5582
6322
8077
10702
11197
12533
13104
3043.0
8963.8
14884.6
20805.4
26726.2
32647.0

Figure S2: SDS-PAGE of fraction 47.

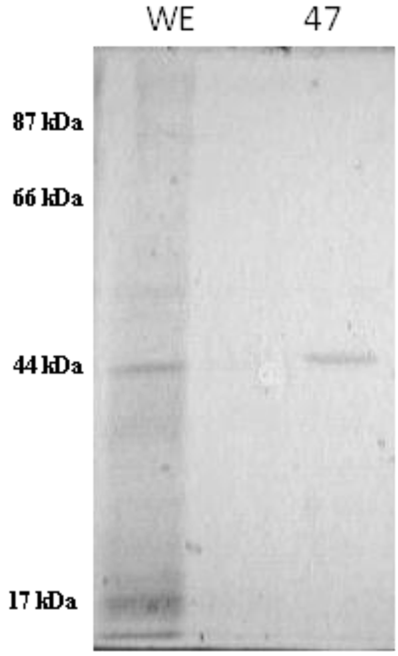

WE: whole extract.

Table S1. Predicted subcellular localization of the 78 identified proteins.

***** not determined; S secreted, C chloroplast, Chlo: chloroplast, CM: cell membrane, Cyto: cytoplasm, ER: endoplasmic reticulum, N: nucleus, P: peroxisomes, V: vacuole. CW: cell wall, y: yes

		Fasta Header	*mGOASVM-Loc*	*TargetP* **Loc**	*SignalP (Sec/SPI)*	*ChloroP*	*WallProtDB*
1.		tr|A0A067FXS4|A0A067FXS4_CITSI Alanine--tRNA ligase OS=Citrus sinensis OX=2711 GN=CISIN_1g002252mg PE=3 SV=1	*	*	*	*	*
2.		tr|A0A067FQ29|A0A067FQ29_CITSI Probable alanine--tRNA ligase, chloroplastic OS=Citrus sinensis OX=2711 GN=CISIN_1g002252mg PE=3 SV=1	*	*	*	*	*
3.		tr|A0A067FS06|A0A067FS06_CITSI Uncharacterized protein OS=Citrus sinensis OX=2711 GN=CISIN_1g0008471mg PE=4 SV=1	*	**S**	*	*	*
4.		tr|A0A067GUC9|A0A067GUC9_CITSI Uncharacterized protein OS=Citrus sinensis OX=2711 GN=CISIN_1g023596mg PE=4 SV=1	*	**C**	*	**y**	*
5.		tr|A0A067FLL5|A0A067FLL5_CITSI Alanine--tRNA ligase OS=Citrus sinensis OX=2711 GN=CISIN_1g002252mg PE=3 SV=1	*	*	*	*	*
6.		tr|A0A067FBM6|A0A067FBM6_CITSI Uncharacterized protein OS=Citrus sinensis OX=2711 GN=CISIN_1g026103mg PE=4 SV=1	*	**S**	*	**y**	*
7.		tr|A0A067EPP0|A0A067EPP0_CITSI Uncharacterized protein (Fragment) OS=Citrus sinensis OX=2711 GN=CISIN_1g043053mg PE=4 SV=1	*	*	*	*	**cw**
8.		tr|A0A067DXK8|A0A067DXK8_CITSI Uncharacterized protein OS=Citrus sinensis OX=2711 GN=CISIN_1g007928mg PE=4 SV=1	*	*	*	*	*
9.		tr|A0A067E608|A0A067E608_CITSI Uncharacterized protein OS=Citrus sinensis OX=2711 GN=CISIN_1g007928mg PE=4 SV=1	*	*	*	*	*
10.		tr|A0A067FZS8|A0A067FZS8_CITSI Uncharacterized protein OS=Citrus sinensis OX=2711 GN=CISIN_1g005316mg PE=4 SV=1	*	*	*	*	*
11.		tr|A0A067G6L7|A0A067G6L7_CITSI Uncharacterized protein OS=Citrus sinensis OX=2711 GN=CISIN_1g0022182mg PE=4 SV=1	*	*	*	*	*
12.		tr|A0A067G9E6|A0A067G9E6_CITSI Uncharacterized protein OS=Citrus sinensis OX=2711 GN=CISIN_1g0022182mg PE=4 SV=1	*	*	*	*	*
13.		tr|A0A067GBI2|A0A067GBI2_CITSI Uncharacterized protein OS=Citrus sinensis OX=2711 GN=CISIN_1g005316mg PE=4 SV=1	*	*	*	*	*
14.		tr|A0A067GIK6|A0A067GIK6_CITSI Uncharacterized protein OS=Citrus sinensis OX=2711 GN=CISIN_1g0022182mg PE=4 SV=1	*	*	*	*	*
15.		tr|A0A067GIV0|A0A067GIV0_CITSI Uncharacterized protein OS=Citrus sinensis OX=2711 GN=CISIN_1g0022182mg PE=4 SV=1	*	*	*	*	*
16.		tr|A0A067GUN6|A0A067GUN6_CITSI Uncharacterized protein OS=Citrus sinensis OX=2711 GN=CISIN_1g023596mg PE=4 SV=1	*	C	*	y	*
17.		tr|A0A067GDZ1|A0A067GDZ1_CITSI RING-type E3 ubiquitin transferase OS=Citrus sinensis OX=2711 GN=CISIN_1g038217mg PE=4 SV=1	*	*	*	*	*
18.		tr|A0A067H3Y3|A0A067H3Y3_CITSI Uncharacterized protein OS=Citrus sinensis OX=2711 GN=CISIN_1g041151mg PE=4 SV=1	*	*	*	*	*
19.		tr|A0A067EJ84|A0A067EJ84_CITSI Uncharacterized protein OS=Citrus sinensis OX=2711 GN=CISIN_1g019479mg PE=3 SV=1	Chlo	**C**	*	**y**	*
20.		tr|A0A067GV48|A0A067GV48_CITSI Uncharacterized protein OS=Citrus sinensis OX=2711 GN=CISIN_1g045426mg PE=4 SV=1	Chlo	*	*	*	*
21.		tr|C6KK63|C6KK63_CITAR RpoB (Fragment) OS=Citrus aurantium OX=43166 GN=rpoB PE=4 SV=1	Chlo	*	*	*	*
22.		tr|A7U3F5|A7U3F5_CITAR RNA polymerase B (Fragment) OS=Citrus aurantium OX=43166 GN=rpoB2 PE=4 SV=1	Chlo	*	*	*	*
23.		tr|A0A067ECH7|A0A067ECH7_CITSI Uncharacterized protein OS=Citrus sinensis OX=2711 GN=CISIN_1g042086mg PE=4 SV=1	*	**S**	*	**y**	**cw**
24.	1	tr|A0A067DDE4|A0A067DDE4_CITSI Uncharacterized protein OS=Citrus sinensis OX=2711 GN=CISIN_1g012745mg PE=4 SV=1	CM	*	*	*	**cw**
25.	2	tr|A0A067DV99|A0A067DV99_CITSI Uncharacterized protein OS=Citrus sinensis OX=2711 GN=CISIN_1g001084mg PE=4 SV=1	CM	*	*	*	*
26.		tr|A0A067DZ88|A0A067DZ88_CITSI Uncharacterized protein OS=Citrus sinensis OX=2711 GN=CISIN_1g001084mg PE=4 SV=1	CM	*	*	*	*
27.		tr|A0A067ES66|A0A067ES66_CITSI Uncharacterized protein OS=Citrus sinensis OX=2711 GN=CISIN_1g009995mg PE=4 SV=1	CM	*	*	*	*
28.		tr|A0A067EVC3|A0A067EVC3_CITSI Uncharacterized protein OS=Citrus sinensis OX=2711 GN=CISIN_1g023376mg PE=4 SV=1	CM	*	*	*	*
29.		tr|A0A067FYX5|A0A067FYX5_CITSI Uncharacterized protein OS=Citrus sinensis OX=2711 GN=CISIN_1g010129mg PE=3 SV=1	*	**S**	**y**	*	**cw**
30.		tr|A0A067DW09|A0A067DW09_CITSI Uncharacterized protein (Fragment) OS=Citrus sinensis OX=2711 GN=CISIN_1g038402mg PE=4 SV=1	CM	*	*	*	*
31.		tr|A0A067DYR7|A0A067DYR7_CITSI Uncharacterized protein OS=Citrus sinensis OX=2711 GN=CISIN_1g017201mg PE=4 SV=1	*	**S**	*	*	*
32.		tr|A0A067GQ70|A0A067GQ70_CITSI Uncharacterized protein (Fragment) OS=Citrus sinensis OX=2711 GN=CISIN_1g0398371mg PE=4 SV=1	*	**S**	*	*	*
33.		tr|A0A067EAN5|A0A067EAN5_CITSI Uncharacterized protein OS=Citrus sinensis OX=2711 GN=CISIN_1g017201mg PE=3 SV=1	Cyto	*	*	*	*
34.		tr|Q3HM93|Q3HM93_CITSI Glutathione S-transferase OS=Citrus sinensis OX=2711 PE=2 SV=1	*	**S**	*	*	*
35.		tr|A0A067EE86|A0A067EE86_CITSI Uncharacterized protein OS=Citrus sinensis OX=2711 GN=CISIN_1g025336mg PE=4 SV=1	Cyto	*	*	*	*
36.		tr|A0A067DYD3|A0A067DYD3_CITSI Uncharacterized protein OS=Citrus sinensis OX=2711 GN=CISIN_1g017201mg PE=4 SV=1	Cyto	*	*	*	*
37.		tr|A0A067EAX4|A0A067EAX4_CITSI Uncharacterized protein (Fragment) OS=Citrus sinensis OX=2711 GN=CISIN_1g042204mg PE=4 SV=1	Cyto	*	*	*	*
38.		tr|A0A067EGL9|A0A067EGL9_CITSI Uncharacterized protein (Fragment) OS=Citrus sinensis OX=2711 GN=CISIN_1g0107352mg PE=3 SV=1	Cyto	*	*	*	*
39.		tr|A0A067F275|A0A067F275_CITSI Uncharacterized protein OS=Citrus sinensis OX=2711 GN=CISIN_1g041226mg PE=3 SV=1	*	S	*	*	*
40.		tr|A0A067G2U9|A0A067G2U9_CITSI Uncharacterized protein OS=Citrus sinensis OX=2711 GN=CISIN_1g011461mg PE=4 SV=1	Cyto	*	*	*	
41.		tr|A0A067G2Z9|A0A067G2Z9_CITSI Uncharacterized protein OS=Citrus sinensis OX=2711 GN=CISIN_1g011461mg PE=4 SV=1	Cyto	*	*	*	*
42.		tr|A0A067GET1|A0A067GET1_CITSI Uncharacterized protein OS=Citrus sinensis OX=2711 GN=CISIN_1g011461mg PE=4 SV=1	Cyto	*	*	*	*

43.	tr|A0A067GNR1|A0A067GNR1_CITSI Uncharacterized protein OS=Citrus sinensis OX=2711 GN=CISIN_1g000014mg PE=4 SV=1	Cyto	*	*	*	*
44.	tr|A0A067GRF1|A0A067GRF1_CITSI Uncharacterized protein OS=Citrus sinensis OX=2711 GN=CISIN_1g000014mg PE=4 SV=1	Cyto	*	*	*	*
45.	tr|A0A067H0N2|A0A067H0N2_CITSI Uncharacterized protein OS=Citrus sinensis OX=2711 GN=CISIN_1g000014mg PE=4 SV=1	Cyto	*	*	*	*
46.	tr|A0A067GNF9|A0A067GNF9_CITSI Uncharacterized protein OS=Citrus sinensis OX=2711 GN=CISIN_1g000014mg PE=4 SV=1	Cyto	*	*	*	*
47.	tr|A0A067DDS7|A0A067DDS7_CITSI Uncharacterized protein (Fragment) OS=Citrus sinensis OX=2711 GN=CISIN_1g037314mg PE=4 SV=1	Cyto	*	*	*	*
48.	tr|A0A067ECD2|A0A067ECD2_CITSI Uncharacterized protein OS=Citrus sinensis OX=2711 GN=CISIN_1g026596mg PE=4 SV=1	Cyto	*	*	*	*
49.	tr|A0A067EKU4|A0A067EKU4_CITSI SAND domain-containing protein OS=Citrus sinensis OX=2711 GN=CISIN_1g026596mg PE=4 SV=1	Cyto	*	*	*	*
50.	tr|A0A067GCY0|A0A067GCY0_CITSI Uncharacterized protein (Fragment) OS=Citrus sinensis OX=2711 GN=CISIN_1g039101mg PE=4 SV=1	Cyto	*	*	*	*
51.	tr|A0A067DVX6|A0A067DVX6_CITSI Uncharacterized protein OS=Citrus sinensis OX=2711 GN=CISIN_1g014537mg PE=3 SV=1	*	**S**	**y**	*	*
52.	tr|A0A067DIT7|A0A067DIT7_CITSI Uncharacterized protein OS=Citrus sinensis OX=2711 GN=CISIN_1g014537mg PE=3 SV=1	*	**S**	**y**	*	*
53.	tr|A0A067EBP6|A0A067EBP6_CITSI Uncharacterized protein OS=Citrus sinensis OX=2711 GN=CISIN_1g017684mg PE=3 SV=1	*	**S**	**y**	*	**cw**
54.	tr|A0A067EBA9|A0A067EBA9_CITSI Uncharacterized protein OS=Citrus sinensis OX=2711 GN=CISIN_1g017684mg PE=3 SV=1	*	**S**	**y**	*	**cw**
55.	tr|A0A067EF15|A0A067EF15_CITSI Uncharacterized protein OS=Citrus sinensis OX=2711 GN=CISIN_1g017684mg PE=3 SV=1	*	**S**	**y**	*	**cw**
56.	tr|V4TXR3|V4TXR3_9ROSI Uncharacterized protein OS=Citrus clementina OX=85681 GN=CICLE_v10020515mg PE=3 SV=1	*	**S**	*	**y**	**cw**
57.	tr|A0A067DUQ6|A0A067DUQ6_CITSI Uncharacterized protein OS=Citrus sinensis OX=2711 GN=CISIN_1g014537mg PE=3 SV=1	*	**S**	**y**	*	**cw**
58.	tr|A0A067ENI5|A0A067ENI5_CITSI Uncharacterized protein OS=Citrus sinensis OX=2711 GN=CISIN_1g017684mg PE=3 SV=1	*	**S**	*	*	**cw**
59.	tr|A0A067EMQ7|A0A067EMQ7_CITSI Uncharacterized protein OS=Citrus sinensis OX=2711 GN=CISIN_1g017181mg PE=3 SV=1	*	**S**	*	*	*
60.	tr|A0A067FW02|A0A067FW02_CITSI Uncharacterized protein OS=Citrus sinensis OX=2711 GN=CISIN_1g011600mg PE=3 SV=1	*	**S**	**y**	*	**cw**
61.	tr|A0A067FVB0|A0A067FVB0_CITSI Uncharacterized protein OS=Citrus sinensis OX=2711 GN=CISIN_1g011600mg PE=3 SV=1	*	**S**	*	*	**cw**
62.	tr|A0A067DMF5|A0A067DMF5_CITSI Uncharacterized protein OS=Citrus sinensis OX=2711 GN=CISIN_1g014537mg PE=3 SV=1	*	**S**	**Y**	*	**cw**
63.	tr|A0A067EZE8|A0A067EZE8_CITSI Uncharacterized protein (Fragment) OS=Citrus sinensis OX=2711 GN=CISIN_1g0026731mg PE=4 SV=1	ER	**C**	*	**y**	*
64.	tr|A0A067F884|A0A067F884_CITSI Non-specific serine/threonine protein kinase OS=Citrus sinensis OX=2711 GN=CISIN_1g013522mg PE=3 SV=1	ER	*	*	*	*
65.	tr|A0A067DZ15|A0A067DZ15_CITSI Uncharacterized protein (Fragment) OS=Citrus sinensis OX=2711 GN=CISIN_1g043866mg PE=4 SV=1	ER	*	*	*	*
66.	tr|A0A067DAD8|A0A067DAD8_CITSI Uncharacterized protein OS=Citrus sinensis OX=2711 GN=CISIN_1g040817mg PE=4 SV=1	N	*	*	*	*
67.	tr|A0A067EJ07|A0A067EJ07_CITSI Uncharacterized protein OS=Citrus sinensis OX=2711 GN=CISIN_1g020996mg PE=4 SV=1	N	*	*	*	*
68.	tr|A0A067GIB5|A0A067GIB5_CITSI Uncharacterized protein OS=Citrus sinensis OX=2711 GN=CISIN_1g023721mg PE=4 SV=1	N	*	*	*	*
69.	tr|A0A067GQL4|A0A067GQL4_CITSI Uncharacterized protein OS=Citrus sinensis OX=2711 GN=CISIN_1g006427mg PE=4 SV=1	N	*	*	*	*
70.	tr|A0A067GT43|A0A067GT43_CITSI Uncharacterized protein OS=Citrus sinensis OX=2711 GN=CISIN_1g014532mg PE=4 SV=1	N	*	*	*	*
71.	tr|A0A067GVN8|A0A067GVN8_CITSI Uncharacterized protein OS=Citrus sinensis OX=2711 GN=CISIN_1g023721mg PE=4 SV=1	N	*	*	*	*
72.	tr|A0A067GYR1|A0A067GYR1_CITSI Uncharacterized protein OS=Citrus sinensis OX=2711 GN=CISIN_1g003828mg PE=4 SV=1	N	*	*	*	*
73.	tr|A0A067FVE2|A0A067FVE2_CITSI Uncharacterized protein (Fragment) OS=Citrus sinensis OX=2711 GN=CISIN_1g0008471mg PE=4 SV=1	N	*	*	*	*
74.	tr|A0A067G352|A0A067G352_CITSI Uncharacterized protein (Fragment) OS=Citrus sinensis OX=2711 GN=CISIN_1g0008471mg PE=4 SV=1	N	*	*	*	*
75.	tr|A0A067ED32|A0A067ED32_CITSI Uncharacterized protein (Fragment) OS=Citrus sinensis OX=2711 GN=CISIN_1g0173161mg PE=4 SV=1	P	*	*	*	*
76.	tr|A0A067H5U9|A0A067H5U9_CITSI Sodium/hydrogen exchanger OS=Citrus sinensis OX=2711 GN=CISIN_1g009645mg PE=3 SV=1	V	*	*	*	*
77.	tr|A0A067FNX1|A0A067FNX1_CITSI Uncharacterized protein OS=Citrus sinensis OX=2711 GN=CISIN_1g036221mg PE=4 SV=1	V	*	*	*	*
78.	tr|A0A067DCQ1|A0A067DCQ1_CITSI Uncharacterized protein (Fragment) OS=Citrus sinensis OX=2711 GN=CISIN_1g044944mg PE=4 SV=1	V	*	*	*	*

Table S2: PROSITE output for the 20 proteins localized in the secretory pathway.

1	**tr-A0A067FYX5-A0A067FYX5_CITSI**	
	PS51767, PEPTIDASE_A1 Peptidase family A1 Domain: 91 – 394	**Predicted features:** DOMAIN: 91-394; Peptidase A1; ACT_SITE: 109 [group: 1]; ACT_SITE: 304 [group: 1]<br>**Absent feature:** DISULFID: 391-394
	PS51257, PROKAR_LIPOPROTEIN Prokaryotic membrane Lipoprotein, lipid attachment Site: 1 - 19:	**Predicted features:** SIGNAL: 1 - 18[condition: none]; LIPID: 19, N-palmitoyl cysteine<br>LIPID: 19, S-diacylglycerol cysteine
	PS51767, PEPTIDASE_A1 Peptidase family A1; Domain: 105 - 387	**Predicted features:** DOMAIN: 105 -387; Peptidase A1 ACT_SITE: 123; [group: 1]; ACT_SITE: 328; [group: 1]<br>**Absent feature:** DISULFID: 373 -387
	PS00141 ASP_PROTEASE Eukaryotic and viral aspartyl proteases active site: 120 - 131	**Predicted feature:** ACT_SITE: 123; ACT_SITE: 325 – 336; **Predicted feature:** ACT_SITE: 328
2	**tr-A0A067DVX6-A0A067DVX6_CITSI**	
	PS51767 PEPTIDASE_A1 Peptidase family A1, Domain: 91 - 374	**Predicted feature:** DOMAIN: 91 -374, Peptidase A1; **Absent features:** ACT_SITE: 109 [group: 1]; CT_SITE: 318 [group: 1]; DISULFID: 371- 374
3	**tr-A0A067DIT7-A0A067DIT7_CITSI**	
	PS51767 PEPTIDASE_A1, Peptidase family A1, Domain: 91 - 416	**Predicted features:** DOMAIN: 91- 416, Peptidase A1; ACT_SITE: 109, [group: 1]; ACT_SITE: 304, [group: 1]<br>**Absent feature:** DISULFID: 346 -381
4	**tr-A0A067FW02-A0A067FW02_CITSI**	
	PS51767 PEPTIDASE_A1 Peptidase family A1, Domain: 140 – 476	ACT_SITE: 158, [group: 1] ; ACT_SITE: 359; [group: 1], DISULFID: 398 - 439
5	**tr-A0A067DMF5-A0A067DMF5_CITSI**	
	PS51767 PEPTIDASE_A1 Peptidase family A1, Domain: 91 - 345	**Predicted feature:** DOMAIN: 91 – 345, Peptidase A1<br>**Absent features:** ACT_SITE: 109, [group: 1]; ACT_SITE: 339, [group: 1], DISULFID: 345
6	**tr-A0A067DUQ6-A0A067DUQ6_CITSI**	
	PS00141 ASP_PROTEASE Eukaryotic and viral aspartyl proteases active site: 106 - 117	**Predicted feature:** ACT_SITE: 109
7	**tr-A0A067DVX6-A0A067DVX6_CITSI**	
	PS00141 ASP_PROTEASE Eukaryotic and viral aspartyl proteases active site: 106 - 117	**Predicted feature:** ACT_SITE: 109; 252 – 263<br>Predicted feature: ACT_SITE 255
8	**tr-A0A067DIT7-A0A067DIT7_CITSI**	
	PS00141 ASP_PROTEASE Eukaryotic and viral aspartyl proteases, active site: 106 - 117	**Predicted feature:** ACT_SITE: 109
	PS00141 ASP_PROTEASE Eukaryotic and viral aspartyl proteases, active site: 301 - 312	:<br>**Predicted feature:** ACT_SITE: 304
9	**tr-Q3HM93-Q3HM93_CITSI**	
	PS50404 GST_NTER Soluble glutathione S-transferase N-terminal Domain: 1 - 82	**Predicted feature:** DOMAIN: 1-82, GST N-terminal
	PS50405 GST_CTER Soluble glutathione S-transferase C-terminal Domain profile: 89 - 213	**Predicted feature:** DOMAIN: 89 -213, GST C-terminal
10	**tr-A0A067F275-A0A067F275_CITSI**	
	PS50404 GST_NTER Soluble glutathione S-transferase N-terminal Domain: 1 - 82:	**Predicted feature:** DOMAIN: 1- 82, GST N-terminal
	PS50405 GST_CTER Soluble glutathione S-transferase C-terminal Domain: 89 - 213	**Predicted feature:**<br>DOMAIN: 89 -213, GST C-terminal
11	**tr-A0A067FVB0-A0A067FVB0_CITSI**	
	PS51767 PEPTIDASE_A1 Peptidase family A1, Domain: 113 - 449:	**Predicted features:** DOMAIN: 113 -449, Peptidase A1; ACT_SITE:131 [group: 1]; ACT_SITE:332 [group: 1]; DISULFID: 371 -412[condition: C-x*-C]
	PS00141 ASP_PROTEASE Eukaryotic and viral aspartyl proteases Active site: 128 - 139:	**Predicted feature:** ACT_SITE 131
	PS00217 SUGAR_TRANSPORT_2 Sugar transport proteins signature 2<br>Active site: 193 - 218	
12	**tr-A0A067FBM6-A0A067FBM6_CITSI**	
	PS51032 AP2_ERF AP2/ERF; AP2_ERF AP2/ERF;domain: 66 - 123:	
13	**tr-A0A067ECH7-A0A067ECH7_CITSI**	
	PS50011 PROTEIN_KINASE_DOM Protein kinase; domain :517 - 784	**Predicted features:** DOMAIN: 517 -784, Protein kinase; NP_BIND: 523 -531, ATP, [condition: x*], [group: 1]; BINDING: 545, ATP, [condition: K], [group: 1]; ACT_SITE: 645, Proton acceptor [condition: D and <grp:1>]
	PS00109 PROTEIN_KINASE_TYR Tyrosine protein kinases specific; active-site signature: 641 - 653:	**Predicted feature:** ACT_SITE: 645, Proton acceptor
14	**tr-A0A067EPP0-A0A067EPP0_CITSI**	

	PS51450 LRR Leucine-rich repeat LRR Leucine-rich repeat	
	PS50011 PROTEIN_KINASE_DOM Protein kinase domain	**Predicted features:**<br>DOMAIN: 706-992, Protein kinase<br>NP_BIND: 712 -720, ATP, [group: 1]<br>BINDING: 734, ATP [condition: K], [group: 1]<br>ACT_SITE: 841, Proton acceptor, condition: D and <grp:1>]
	PS00107 PROTEIN_KINASE_ATP Protein kinases ATP-binding region signature: 712 - 735:	
	PS00108 PROTEIN_KINASE_ST Serine/Threonine protein kinases active-site signature:<br>837 - 849	**Predicted feature:**<br>ACT_SITE 841, Proton acceptor
15	**tr-A0A067DDE4-A0A067DDE4_CITSI**	
	PS50011 PROTEIN_KINASE_DOM Protein kinase; domain profile: 337 - 457	**Predicted features:** DOMAIN: 337-457, Protein kinase<br>NP_BIND: 343-351, ATP:[condition: x*],[group: 1]<br>BINDING: 365, ATP: [condition: K], [group: 1]<br>**Absent feature:** ACT_SITE: 457, Proton acceptor, [condition not true: D and <grp:1>]
16	*tr-V4TXR3-V4TXR3_9ROSI*	
	PS00008 MYRISTYL N-myristoylation site: 38 - 43: GLvmAL; 112 - 117: GSepTL; 132 - 137: GAnfAS; 250 - 255: GCvpAE; 263 - 268: QcaAD; 332 - 337: GLglCT	**Predicted feature:**MOD_RES, 63, Phosphoserine, [condition: S]<br>214 - 217 SlpD
	PS00006 CK2_PHOSPHO_SITE Casein kinase II phosphorylation site: 63 - 66: SlvD	**Predicted feature:** MOD_RES 214, Phosphoserine, [condition: S]
	PS00005 PKC_PHOSPHO_SITE Protein kinase C phosphorylation site: 76 - 78: TaR	**Predicted feature:** MOD_RES: 76, Phosphothreonine, [condition: T]; 90 - 92: TrR<br>**Predicted feature:** MOD_RES: 90, Phosphothreonine, [condition: T];94 - 96: TgR<br>**Predicted feature:** MOD_RES 94, Phosphothreonine, [condition: T]; 207 - 209: SaR<br>**Predicted feature:** MOD_RES: 207; hosphoserine[condition: S]; 301 - 303: TgK<br>**Predicted feature:** MOD_RES: 301, Phosphothreonine; [condition: T]; 319 - 321: TsK<br>**Predicted feature:** MOD_RES 319, Phosphothreonine; [condition: T]; 358 - 360: SeR<br>**Predicted feature:** MOD_RES 358; Phosphoserine [condition: S]
	PS00004 CAMP_PHOSPHO_SITE cAMP- and cGMP-dependent protein kinase phosphorylation site91 - 94: RRpT	
	PS00001 ASN_GLYCOSYLATION N-glycosylation site: 144 – 147, NDTG CARBOHYD 144 N-linked (GlcNAc	.)<br>**Predicted feature:** asparagine, [condition: N] 381 - 384: NLST<br>**Predicted feature:**CARBOHYD, 381, N-linked (GlcNAc...) asparagine, [condition: N]
17	**tr-A0A067EBP6-A0A067EBP6_CITS**	
	PS00008 MYRISTYL N-myristoylation site14 - 19: GLvmAL; 88 - 93: GSepTL; 108 - 113: GAnfAS; 226 - 231: GCvpAE;239 - 244: GQcaAD; 308 - 313: GLglCT	
	PS00006 CK2_PHOSPHO_SITE Casein kinase II phosphorylation site: 39 - 42: SlvD	**Predicted feature:** MOD_RES: 39; Phosphoserine; [condition: S]; 190 - 193 SlpD<br>**Predicted feature:** MOD_RES: 190; Phosphoserine, [condition: S]
	PS00005 PKC_PHOSPHO_SITE Protein kinase C phosphorylation site: 52 - 54: TaR	**Predicted feature:** MOD_RES: 52, Phosphothreonine, condition: T] 66 – 68: TrR<br>**Predicted feature:** MOD_RES: 66, Phosphothreonine, [condition: T] 70 - 72: TgR<br>**Predicted feature:**MOD_RES 70, Phosphothreonine, [condition: T] 183 - 185: SaR<br>**Predicted feature:**MOD_RES 183, Phosphoserine [condition: S] 277 - 279: TgK<br>**Predicted feature:** MOD_RES 277, Phosphothreonine, [condition: T]; 295 - 297: TsK<br>**Predicted feature:** MOD_RES: 295, Phosphothreonine, [condition: T]; 334 - 336: SeR<br>**Predicted feature:** MOD_RES 334, Phosphoserine, [condition: S]
	PS00004 CAMP_PHOSPHO_SITE cAMP- and cGMP-dependent protein kinase phosphorylation site: 67 - 70: RRpT	
	PS00001 ASN_GLYCOSYLATION N-glycosylation site:<br>120 - 123: NDTG	**Predicted feature:** CARBOHYD 120 N-linked (GlcNAc...) asparagine [condition: N],<br>357 - 360: NLST<br>**Predicted feature:** CARBOHYD 357, N-linked (GlcNAc...) asparagine, [condition: N]
18	**tr-A0A067EBA9-A0A067EBA9_CITSI**	

	PS00008 MYRISTYL N-myristoylation site : 14 - 19: GLvmAL;<br>201 - 206: GCvpAE; 214 - 219: GQcaAD; 283 - 288: GLglCT	
	PS00006 CK2_PHOSPHO_SITE Casein kinase II phosphorylation site :39 - 42: SlvD	**Predicted feature:** MOD_RES. 39, Phosphoserine, [condition: S]; 165 - 168: SlpD<br>**Predicted feature:** MOD_RES: 165, Phosphoserine, [condition: S]
	PS00005 PKC_PHOSPHO_SITE Protein kinase C phosphorylation site: 52 - 54: TaR	**Predicted feature:** MOD_RES 52, Phosphothreonine, [condition: T] 66 - 68: TrR<br>**Predicted feature:** MOD_RES. 66, Phosphothreonine, [condition: T]; 70 - 72: TgR<br>**Predicted feature:** MOD_RES: 70, Phosphothreonine, [condition: T] 158 - 160: SaR<br>**Predicted feature:** MOD_RES: 158, Phosphoserine, [condition: S]; 252 - 254: TgK<br>**Predicted feature:** MOD_RES: 252, Phosphothreonine, [condition: T]; 270 - 272: TsK<br>**Predicted feature:** MOD_RES: 270, Phosphothreonine, [condition: T] 309 - 311: SeR;<br>**Predicted feature:**<br>MOD_RES 309, Phosphoserine, [condition: S]
	PS00004 CAMP_PHOSPHO_SITE cAMP- and cGMP-dependent protein kinase phosphorylation site:<br>67 - 70: RRpT	
	PS00001 ASN_GLYCOSYLATION N-glycosylation site:<br>95 - 98 NDTG	**Predicted feature:** CARBOHYD 95, N-linked (GlcNAc...) asparagine, [condition: N]; 332 - 335 NLST<br>**Predicted feature:** CARBOHYD, 332, N-linked (GlcNAc...) asparagine [condition: N]
19	**tr-A0A067EF15-A0A067EF15_CITSI**	
	PS00008 MYRISTYL N-myristoylation site:<br>14 - 19: GLvmAL; 88 - 93: GSepTL; 108 - 113: GAnfAS; 226 - 231: GCvpAE; 239 - 244: GQcaAD;	
	PS00006 CK2_PHOSPHO_SITE Casein kinase II phosphorylation site: 39 - 42: SlvD	**Predicted feature:** MOD_RES:39: Phosphoserine; [condition: S]; 190 - 193: SlpD;<br>**Predicted feature:** MOD_RES:190; Phosphoserine; [condition: S]
	PS00005 PKC_PHOSPHO_SITE Protein kinase C phosphorylation site: 52 - 54: TaR	**Predicted feature:** MOD_RES: 52 Phosphothreonine; [condition: T]; 66 - 68: TrR<br>**Predicted feature**: MOD_RES:66; Phosphothreonine,[condition: T]<br>70 - 72: TgR; Predicted feature: MOD_RES: 70; phosphothreonine; [condition: T] 183 - 185: SaR<br>**Predicted feature:**<br>MOD_RES 183; Phosphoserine; [condition: S]<br>277 - 279: TgK; Predicted feature: MOD_RES; 277; Phosphothreonine, [condition: T] 315 - 317: SnK<br>**Predicted feature:**<br>MOD_RES 315; Phosphoserine; [condition: S]
	PS00004 CAMP_PHOSPHO_SITE cAMP- and cGMP-dependent protein kinase phosphorylation site: 67 - 70: RRpT	
	PS00001 ASN_GLYCOSYLATION N-glycosylation site: 120 - 123: NDTG	Predicted feature: CARBOHYD: 120; N-linked (GlcNAc...) asparagine; [condition: N] 316 - 319: NKSI<br>**Predicted feature:** CARBOHYD: 316; N-linked (GlcNAc...) asparagine; [condition: N]
20	tr-A0A067ENI5-A0A067ENI5_CITSI	
	PS00008 MYRISTYL N-myristoylation site: 8 - 13: GQhiGS<br>12 - 17: GSepTL; 32 - 37: GAnfAS; 150 - 155: GCvpAE; 163 - 168: GQcaAD; 232 - 237: GLglCT	
	PS00001 ASN_GLYCOSYLATION N-glycosylation site:<br>44 - 47: NDTG	**Predicted feature:**<br>CARBOHYD: 44; N-linked (GlcNAc...) asparagine; [condition: N]; 281 - 284: NLST<br>**Predicted feature:**<br>CARBOHYD: 281; N-linked (GlcNAc...) asparagine;[condition: N]
	PS00005 PKC_PHOSPHO_SITE Protein kinase C phosphorylation site: 107 - 109: SaR	**Predicted feature:**<br>MOD_RES: 107; Phosphoserine; [condition: S]; 201 - 203: TgK<br>**Predicted feature:**<br>MOD_RES 201; Phosphothreonine; [condition: T]; 219 - 221: TsK<br>**Predicted feature:**<br>MOD_RES 219; Phosphothreonine; [condition: T]; 258 - 260: SeR<br>PreDicted feature: MOD_RES: 258; Phosphoserine; [condition: S]
	PS00006 CK2_PHOSPHO_SITE Casein kinase II phosphorylation site: 114 - 117: SlpD	**Predicted feature:** MOD_RES:114; Phosphoserine; [condition: S]
	**tr-A0A067EMQ7-A0A067EMQ7_CITSI**	
	PS00008 MYRISTYL N-myristoylation site : 22 - 27: GIafAL<br>115 - 120: GAnfAS; 158 - 163: GAqqAR; 233 - 238: GCvpAE	

	243 - 248: GSngGC; 246 - 251: GGcsAE; 309 - 314: GQgpNN<br>315 - 320: GLglCT; 317 - 322: GLctAL	
	PS00006 CK2_PHOSPHO_SITE Casein kinase II phosphorylation site: 45 - 48: SlvD	Predicted feature: MOD_RES: 45; Phosphoserine; [condition: S]; 287 - 290: ThmD;<br>Predicted feature: MOD_RES: 287; Phosphothreonine; [condition: T]
	PS00005 PKC_PHOSPHO_SITE Protein kinase C phosphorylation site : 58 - 60: TaR	Predicted feature: MOD_RES: 58; Phosphothreonine; [condition: T]; 72 - 74: ThR<br>Predicted feature: MOD_RES: 72Phosphothreonine; [condition: T]; 76 - 78: TgR;<br>Predicted feature:<br>MOD_RES: 76; Phosphothreonine; [condition: T]; 90 - 92: SqR;<br>Predicted feature: MOD_RES: 90; Phosphoserine; [condition: S]; 190 - 192: SaR<br>Predicted feature: MOD_RES 190; Phosphoserine; [condition: S]; 302 - 304: TaK<br>Predicted feature: MOD_RES 302; Phosphothreonine; [condition: T]; 341 - 343: SeK<br>Predicted feature: MOD_RES 341; Phosphoserine; [condition: S]
	PS00001 ASN_GLYCOSYLATION N-glycosylation site : 127 - 130: NDTG	Predicted feature: CARBOHYD: 127; N-linked (GlcNAc...) asparagine; [condition: N]; 364 - 367: NLST<br>Predicted feature: CARBOHYD: 364; N-linked (GlcNAc...) asparagine; [condition: N]
	PS00004 CAMP_PHOSPHO_SITE cAMP- and cGMP-dependent protein kinase phosphorylation site: 151 - 154: RRvS	

Table S3: K numbers (KO) by BlastKOALA (https://www.kegg.jp/blastkoala/).

	Query	KO	Definition	Score	Second best	
**1**	tr|A0A067FLL5|A0A067FLL5_CITSI	K01872	AARS; alanyl-tRNA synthetase [EC:6.1.1.7]	431		
**2**	tr|A0A067FXS4|A0A067FXS4_CITSI	K01872	AARS; alanyl-tRNA synthetase [EC:6.1.1.7]	431		
**3**	tr|A0A067EAN5|A0A067EAN5_CITSI	K00121	frmA; S-(hydroxymethyl)glutathione dehydrogenase / alcohol dehydrogenase [EC:1.1.1.284 1.1.1.1]	243		
**4**	tr|Q3HM93|Q3HM93_CITSI	K00799	GST; glutathione S-transferase [EC:2.5.1.18]	174		
**5**	tr|A0A067F884|A0A067F884_CITSI			319	K07198	35
**6**	tr|A0A067FQ29|A0A067FQ29_CITSI	K01872	AARS; alanyl-tRNA synthetase [EC:6.1.1.7]	739		
**7**	tr|A0A067EE86|A0A067EE86_CITSI	K00121	frmA; S-(hydroxymethyl)glutathione dehydrogenase / alcohol dehydrogenase [EC:1.1.1.284 1.1.1.1]	125		
**8**	tr|A0A067H5U9|A0A067H5U9_CITSI			287	K14724	71
**9**	tr|A0A067DAD8|A0A067DAD8_CITSI			58		
**10**	tr|A0A067DDE4|A0A067DDE4_CITSI			181		
**11**	tr|A0A067DMF5|A0A067DMF5_CITSI			53	K01381	21
**12**	tr|A0A067DUQ6|A0A067DUQ6_CITSI			146	K01381	35
**13**	tr|A0A067DV99|A0A067DV99_CITSI			381		
**14**	tr|A0A067DVX6|A0A067DVX6_CITSI			102	K01381	44
**15**	tr|A0A067DXK8|A0A067DXK8_CITSI			286		
**16**	tr|A0A067DYD3|A0A067DYD3_CITSI	K00121	frmA; S-(hydroxymethyl)glutathione dehydrogenase / alcohol dehydrogenase [EC:1.1.1.284 1.1.1.1]	132		
**17**	tr|A0A067DYR7|A0A067DYR7_CITSI	K00121	frmA; S-(hydroxymethyl)glutathione dehydrogenase / alcohol dehydrogenase [EC:1.1.1.284 1.1.1.1]	155		
**18**	tr|A0A067DZ88|A0A067DZ88_CITSI			55		
**19**	tr|A0A067E608|A0A067E608_CITSI			285		
**20**	tr|A0A067EAX4|A0A067EAX4_CITSI	K15042	KPNA5_6; importin subunit alpha-6/7	279		
**21**	tr|A0A067ECD2|A0A067ECD2_CITSI			211		
**22**	tr|A0A067ECH7|A0A067ECH7_CITSI			253		
**23**	tr|A0A067EGL9|A0A067EGL9_CITSI	K12355	REF1; coniferyl-aldehyde dehydrogenase [EC:1.2.1.68]	127		
**24**	tr|A0A067EJ07|A0A067EJ07_CITSI			142		
**25**	tr|A0A067EJ84|A0A067EJ84_CITSI	K12502	VTE3; MPBQ/MSBQ methyltransferase [EC:2.1.1.295]	294		
**26**	tr|A0A067ES66|A0A067ES66_CITSI			244		
**27**	tr|A0A067EPP0|A0A067EPP0_CITSI			771		
**28**	tr|A0A067EVC3|A0A067EVC3_CITSI			144		
**29**	tr|A0A067F275|A0A067F275_CITSI	K00799	GST; glutathione S-transferase [EC:2.5.1.18]	172		
**30**	tr|A0A067FBM6|A0A067FBM6_CITSI			142	K09286	35
**31**	tr|A0A067FNX1|A0A067FNX1_CITSI			114		
**32**	tr|A0A067FS06|A0A067FS06_CITSI			367		
**33**	tr|A0A067FYX5|A0A067FYX5_CITSI			177		
**34**	tr|A0A067FZS8|A0A067FZS8_CITSI			332		
**35**	tr|A0A067G2U9|A0A067G2U9_CITSI			367		
**36**	tr|A0A067G2Z9|A0A067G2Z9_CITSI			369		
**37**	tr|A0A067G6L7|A0A067G6L7_CITSI	K20798	HENMT1; small RNA 2'-O-methyltransferase [EC:2.1.1.-]	601		
**38**	tr|A0A067G9E6|A0A067G9E6_CITSI	K20798	HENMT1; small RNA 2'-O-methyltransferase [EC:2.1.1.-]	599		
**39**	tr|A0A067GBI2|A0A067GBI2_CITSI			662		
**40**	tr|A0A067GET1|A0A067GET1_CITSI			395		
**41**	tr|A0A067GIB5|A0A067GIB5_CITSI			61		
**42**	tr|A0A067GIK6|A0A067GIK6_CITSI	K20798	HENMT1; small RNA 2'-O-methyltransferase [EC:2.1.1.-]	574		
**43**	tr|A0A067GIV0|A0A067GIV0_CITSI	K20798	HENMT1; small RNA 2'-O-methyltransferase [EC:2.1.1.-]	396		
**44**	tr|A0A067GNR1|A0A067GNR1_CITSI	K10592	HUWE1; E3 ubiquitin-protein ligase HUWE1 [EC:2.3.2.26]	2840		
**45**	tr|A0A067GQL4|A0A067GQL4_CITSI			395		
**46**	tr|A0A067GRF1|A0A067GRF1_CITSI	K10592	HUWE1; E3 ubiquitin-protein ligase HUWE1 [EC:2.3.2.26]	2667		
**47**	tr|A0A067GT43|A0A067GT43_CITSI			218		
**48**	tr|A0A067GUC9|A0A067GUC9_CITSI			240		
**49**	tr|A0A067GUN6|A0A067GUN6_CITSI			121		
**50**	tr|A0A067GV48|A0A067GV48_CITSI			2	K06847	1
**51**	tr|A0A067GVN8|A0A067GVN8_CITSI			39		
**52**	tr|A0A067GYR1|A0A067GYR1_CITSI			109		
**53**	tr|A0A067H0N2|A0A067H0N2_CITSI	K10592	HUWE1; E3 ubiquitin-protein ligase HUWE1 [EC:2.3.2.26]	2841		
**54**	tr|A0A067H3Y3|A0A067H3Y3_CITSI	K14272	GGAT; glutamate--glyoxylate aminotransferase [EC:2.6.1.4 2.6.1.2 2.6.1.44]	539		
**55**	tr|A0A067GNF9|A0A067GNF9_CITSI	K10592	HUWE1; E3 ubiquitin-protein ligase HUWE1 [EC:2.3.2.26]	2844		
**56**	tr|A0A067DIT7|A0A067DIT7_CITSI			171	K01381	42
**57**	tr|A0A067EBP6|A0A067EBP6_CITSI			386		
**58**	tr|A0A067EBA9|A0A067EBA9_CITSI			320		
**59**	tr|A0A067EF15|A0A067EF15_CITSI			240		
**60**	tr|A0A067ENI5|A0A067ENI5_CITSI			184		
**61**	tr|A0A067EMQ7|A0A067EMQ7_CITSI			339		

**62**	tr|V4TXR3|V4TXR3_9ROSI			343		
**63**	tr|A0A067FW02|A0A067FW02_CITSI			297		
**64**	tr|A0A067FVB0|A0A067FVB0_CITSI			250		
**65**	tr|A0A067DCQ1|A0A067DCQ1_CITSI			254		
**66**	tr|A0A067DDS7|A0A067DDS7_CITSI			70		
**67**	tr|A0A067DZ15|A0A067DZ15_CITSI			209		
**68**	tr|A0A067EZE8|A0A067EZE8_CITSI			329		
**69**	tr|A0A067FVE2|A0A067FVE2_CITSI			601		
**70**	tr|A0A067G352|A0A067G352_CITSI			570		
**71**	tr|A0A067GCY0|A0A067GCY0_CITSI			315		
**72**	tr|A0A067GQ70|A0A067GQ70_CITSI	K01904	4CL; 4-coumarate--CoA ligase [EC:6.2.1.12]	244		
**73**	tr|A0A067ED32|A0A067ED32_CITSI			22	K19398	1
**74**	tr|A0A067DW09|A0A067DW09_CITSI			5	K09749	1
**75**	tr|A0A067EKU4|A0A067EKU4_CITSI			123		
**76**	tr|C6KK63|C6KK63_CITAR					
**77**	tr|A7U3F5|A7U3F5_CITAR					
**78**	tr|A0A067GDZ1|A0A067GDZ1_CITSI			577		

Table S4: KEGG Mapper Search Result

Pathway (18)

- cit01100 Metabolic pathways - Citrus sinensis (Valencia orange) (81)
  ko:K00121 frmA; S-(hydroxymethyl)glutathione dehydrogenase / alcohol dehydrogenase [EC:1.1.1.284 1.1.1.1]
  ko:K00799 GST; glutathione S-transferase [EC:2.5.1.18]
  ko:K01904 4CL; 4-coumarate--CoA ligase [EC:6.2.1.12]
  ko:K12502 VTE3; MPBQ/MSBQ methyltransferase [EC:2.1.1.295]
  ko:K14272 GGAT; glutamate--glyoxylate aminotransferase [EC:2.6.1.4 2.6.1.2 2.6.1.44]
- cit00480 Glutathione metabolism - Citrus sinensis (Valencia orange) (58)
  ko:K00799 GST; glutathione S-transferase [EC:2.5.1.18]
- cit01110 Biosynthesis of secondary metabolites - Citrus sinensis (Valencia orange) (23)
  ko:K00121 frmA; S-(hydroxymethyl)glutathione dehydrogenase / alcohol dehydrogenase [EC:1.1.1.284 1.1.1.1]
  ko:K01904 4CL; 4-coumarate--CoA ligase [EC:6.2.1.12]
  ko:K12502 VTE3; MPBQ/MSBQ methyltransferase [EC:2.1.1.295]
  ko:K14272 GGAT; glutamate--glyoxylate aminotransferase [EC:2.6.1.4 2.6.1.2 2.6.1.44]
- cit01200 Carbon metabolism - Citrus sinensis (Valencia orange) (12)
  ko:K00121 frmA; S-(hydroxymethyl)glutathione dehydrogenase / alcohol dehydrogenase [EC:1.1.1.284 1.1.1.1]
  ko:K14272 GGAT; glutamate--glyoxylate aminotransferase [EC:2.6.1.4 2.6.1.2 2.6.1.44]
- cit00940 Phenylpropanoid biosynthesis - Citrus sinensis (Valencia orange) (11)
  ko:K01904 4CL; 4-coumarate--CoA ligase [EC:6.2.1.12]
  ko:K12355 REF1; coniferyl-aldehyde dehydrogenase [EC:1.2.1.68]
- cit00130 Ubiquinone and other terpenoid-quinone biosynthesis-Citrus sinensis (Valencia orange) (11)
  ko:K01904 4CL; 4-coumarate--CoA ligase [EC:6.2.1.12]
  ko:K12502 VTE3; MPBQ/MSBQ methyltransferase [EC:2.1.1.295]
- cit00010 Glycolysis / Gluconeogenesis - Citrus sinensis (Valencia orange) (11)
  ko:K00121 frmA; S-(hydroxymethyl)glutathione dehydrogenase / alcohol dehydrogenase [EC:1.1.1.284 1.1.1.1]
- cit00071 Fatty acid degradation - Citrus sinensis (Valencia orange) (11)
  ko:K00121 frmA; S-(hydroxymethyl)glutathione dehydrogenase / alcohol dehydrogenase [EC:1.1.1.284 1.1.1.1]
- cit00350 Tyrosine metabolism - Citrus sinensis (Valencia orange) (11)
  ko:K00121 frmA; S-(hydroxymethyl)glutathione dehydrogenase / alcohol dehydrogenase [EC:1.1.1.284 1.1.1.1]
- cit00970 Aminoacyl-tRNA biosynthesis - Citrus sinensis (Valencia orange) (2)
  ko:K01872 AARS; alanyl-tRNA synthetase [EC:6.1.1.7]
- cit04120 Ubiquitin mediated proteolysis - Citrus sinensis (Valencia orange) (2)
  ko:K10592 HUWE1; E3 ubiquitin-protein ligase HUWE1 [EC:2.3.2.26]
- cit00250 Alanine, aspartate and glutamate metabolism - Citrus sinensis (Valencia orange) (1)
  ko:K14272 GGAT; glutamate--glyoxylate aminotransferase [EC:2.6.1.4 2.6.1.2 2.6.1.44]
- cit01230 Biosynthesis of amino acids - Citrus sinensis (Valencia orange) (1)
  ko:K14272 GGAT; glutamate--glyoxylate aminotransferase [EC:2.6.1.4 2.6.1.2 2.6.1.44]
- cit00260 Glycine, serine and threonine metabolism - Citrus sinensis (Valencia orange) (1)
  ko:K14272 GGAT; glutamate--glyoxylate aminotransferase [EC:2.6.1.4 2.6.1.2 2.6.1.44]
- cit00630 Glyoxylate and dicarboxylate metabolism - Citrus sinensis (Valencia orange) (1)
  ko:K14272 GGAT; glutamate--glyoxylate aminotransferase [EC:2.6.1.4 2.6.1.2 2.6.1.44]
- cit01210 2-Oxocarboxylic acid metabolism - Citrus sinensis (Valencia orange) (1)
  ko:K14272 GGAT; glutamate--glyoxylate aminotransferase [EC:2.6.1.4 2.6.1.2 2.6.1.44]
- cit00220 Arginine biosynthesis - Citrus sinensis (Valencia orange) (1)
  ko:K14272 GGAT; glutamate--glyoxylate aminotransferase [EC:2.6.1.4 2.6.1.2 2.6.1.44]
- cit00710 Carbon fixation in photosynthetic organisms - Citrus sinensis (Valencia orange) (1)
  ko:K14272 GGAT; glutamate--glyoxylate aminotransferase [EC:2.6.1.4 2.6.1.2 2.6.1.44]

Table S5. MS/MS identified peptides of GDSLs.

Accession	Start-End	Sequence	m/z
**A0A067EBP6_CITSI**			
	1-10	MASSFVFGVR 1Acetyl 1Oxidation	1158.61
	1-10	MASSFVFGVR 1Acetyl	1142.58
	11-31	TILGLVMALGALAPQAAEAAR 1Oxidation	2053.17
	1-31	MASSFVFGVRTILGLVmALGALAPQAAEAAR 1Oxidation	3134.72
	1-31	MASSFVFGVRTILGLVMALGALAPQAAEAAR 1Acetyl 1Oxidation	3176.73
	146-156	VTALIGPQRTK	1183.73
	187-208	QFSLPDYVKYVISEYRKLLTR	2618.46
	197-215	YVISEYRKLLTRLYDLGAR	2329.33
	216-235	RVLVTGTGPLGCVPAERAMR	2083.15
	216-237	RVLVTGTGPLGCVPAERAmRGR (1Oxidation)	2312.27
	217-235	VLVTGTGPLGCVPAERAMR	1927.04
	233-247	AMRGRNGQCAADLQR	1646.81
	262-289	DLNSQYGSEIFVAVNTGKMQYNFISNPR	3192.57
	55-72	ADSPPYGIDYPTRRPTGR	2019.02
**A0A067EBA9_CITSI**			
	107-131	MFRQFEYFQEYQNRVTALIGPQRTK	3150.63
	1-10	MASSFVFGVR 1Acetyl 1Oxidation	1158.57
	121-131	VTALIGPQRTK	1183.73
	1-31	MASSFVFGVRTILGLVmALGALAPQAAEAAR 1Oxidation	3134.72
	1-31	MASSFVFGVRTILGLVMALGALAPQAAEAAR 1Acetyl 1Oxidation	3176.73
	163-171	QFSLPDYVK	1096.58
	163-183	QFSLPDYVKYVISEYRKLLTR	2618.46
	172-190	YVISEYRKLLTRLYDLGAR	2329.33
	191-210	RVLVTGTGPLGCVPAERAMR	2083.15
	191-212	RVLVTGTGPLGCVPAERAmRGR (1Oxidation)	2312.24
	192-210	VLVTGTGPLGCVPAERAMR	1927.04
	208-222	AMRGRNGQCAADLQR	1646.81
	237-264	DLNSQYGSEIFVAVNTGKMQYNFISNPR	3192.57
	55-72	ADSPPYGIDYPTRRPTGR	2019.02
**A0A067EF15_CITSI**			
	1-10	MASSFVFGVR 1Acetyl 1Oxidation	1158.57
	112-132	QFSLPDYVKYVISEYRKLLTR	2618.46
	1-31	MASSFVFGVRTILGLVmALGALAPQAAEAAR 1Oxidation	3134.72
	1-31	MASSFVFGVRTILGLVMALGALAPQAAEAAR 1Acetyl 1Oxidation	3176.73
	140-161	RVLVTGTGPLGCVPAERAmRGR(1Oxidation)	2312.27
	146-156	VTALIGPQRTK	1183.73
	197-215	YVISEYRKLLTRLYDLGAR	2329.31
	216-235	RVLVTGTGPLGCVPAERAMR	2083.15
	217-235	VLVTGTGPLGCVPAERAMR	1927.04
	233-247	AMRGRNGQCAADLQR	1646.81
	262-289	DLNSQYGSEIFVAVNTGKMQYNFISNPR	3192.57
	306-317	EKIIGDSCCSNK	1296.61
	55-72	ADSPPYGIDYPTRRPTGR	2019.02
**A0A067ENI5_CITSI**			
	112-132	QFSLPDYVKYVISEYRKLLTR	2618.46
	121-139	YVISEYRKLLTRLYDLGAR	2329.31
	140-159	RVLVTGTGPLGCVPAERAMR	2083.15

	140-161	RVLVTGTGPLGCVPAERAmRGR (1Oxidation)	2312.27
	141-159	VLVTGTGPLGCVPAERAMR	1927.04
	157-171	AMRGRNGQCAADLQR	1646.81
	186-213	DLNSQYGSEIFVAVNTGKMQYNFISNPR	3192.57
	70-80	VTALIGPQRTK	1183.73
**A0A067EMQ7_CITSI**			
	1-15	MSMAIATSSASVAMR	1513.73
	153-168	VSAVIGAQQARQLVNR	1709.99
	169-192	ALVLITVGGNDFVNNYYLVPYSAR	2658.42
	195-203	QFTLPNYVK	1109.61
	195-211	QFTLPNYVKYIISEYRK	2162.19
	195-211	QFTLPNYVKYIISEYRK	2162.19
	204-211	YIISEYRK	1071.59
	211-222	KLLmRLYELGAR (1Oxidation)	1478.87
	211-223	KLLmRLYELGARR (1Oxidation)	1634.97
	223-242	RVLVTGTGPLGCVPAELALR	2022.17
	243-254	GSNGGCSAELQR	1179
	61-78	ADAPPYGIDFPTHRPTGR	1967.99
**V4TXR3_9ROSI**			
	1-10	MASSFVFGVR 1Acetyl 1Oxidation	1158.57
	1-17	AVEPWPKLHSKLRFSR 1Acety	1993.13
	1-17	1Met-loss (-)MAVEPWPKLHSKLRFSR	1951.12
	15-34	FSRIRVKYNTMASSFVFGVR	2365.28
	20-34	VKYNTMASSFVFGVR	1705.89
	221-239	YVISEYRKLLTRLHDLGAR	2303.32
	241-259	VLVTGTGPLGCVPAERAMR	1927.04
	257-271	AMRGRNGQCAADLQR	1646.81
**GDL79_ARATH**			
	202-213	KVLRKmYDLGAR	1465.85
	207-235	mYDLGARRVLVTGTGPmGCVPAELAQRSR 2Oxidation	3136.61
	71-102	FSNGLNIPDLISEHLGQESPMPYLSPMLKKDK	3598.83
